# The impact of indoor residual spraying on *Plasmodium falciparum* microsatellite variation in an area of high seasonal malaria transmission in Ghana, West Africa

**DOI:** 10.1111/mec.16029

**Published:** 2021-07-16

**Authors:** Dionne C. Argyropoulos, Shazia Ruybal‐Pesántez, Samantha L. Deed, Abraham R. Oduro, Samuel K. Dadzie, Maxwell A. Appawu, Victor Asoala, Mercedes Pascual, Kwadwo A. Koram, Karen P. Day, Kathryn E. Tiedje

**Affiliations:** ^1^ School of BioSciences Bio21 Institute The University of Melbourne Melbourne Vic. Australia; ^2^ Department of Microbiology and Immunology Bio21 Institute and Peter Doherty Institute The University of Melbourne Melbourne Vic. Australia; ^3^ Navrongo Health Research Centre Ghana Health Service Navrongo Ghana; ^4^ Noguchi Memorial Institute for Medical Research University of Ghana Legon Ghana; ^5^ Department of Ecology and Evolution University of Chicago Chicago USA; ^6^ Present address: Population Health and Immunity Division, Walter and Eliza Hall Institute of Medical Research Melbourne Vic. Australia; ^7^ Present address: Department of Medical Biology and Bio21 Institute The University of Melbourne Melbourne Vic. Australia; ^8^ Present address: Burnet Institute Melbourne Vic. Australia

**Keywords:** genetic epidemiology, indoor residual spraying, malaria elimination, microsatellite genotyping, neutral genetic variation, *Plasmodium falciparum*

## Abstract

Here, we report the first population genetic study to examine the impact of indoor residual spraying (IRS) on *Plasmodium falciparum* in humans. This study was conducted in an area of high seasonal malaria transmission in Bongo District, Ghana. IRS was implemented during the dry season (November–May) in three consecutive years between 2013 and 2015 to reduce transmission and attempt to bottleneck the parasite population in humans towards lower diversity with greater linkage disequilibrium. The study was done against a background of widespread use of long‐lasting insecticidal nets, typical for contemporary malaria control in West Africa. Microsatellite genotyping with 10 loci was used to construct 392 *P*. *falciparum* multilocus infection haplotypes collected from two age‐stratified cross‐sectional surveys at the end of the wet seasons pre‐ and post‐IRS. Three‐rounds of IRS, under operational conditions, led to a >90% reduction in transmission intensity and a 35.7% reduction in the *P*. *falciparum* prevalence (*p* < .001). Despite these declines, population genetic analysis of the infection haplotypes revealed no dramatic changes with only a slight, but significant increase in genetic diversity (*H*_e_: pre‐IRS = 0.79 vs. post‐IRS = 0.81, *p* = .048). Reduced relatedness of the parasite population (*p* < .001) was observed post‐IRS, probably due to decreased opportunities for outcrossing. Spatiotemporal genetic differentiation between the pre‐ and post‐IRS surveys (*D* = 0.0329 [95% CI: 0.0209 – 0.0473], *p* = .034) was identified. These data provide a genetic explanation for the resilience of *P. falciparum* to short‐term IRS programmes in high‐transmission settings in sub‐Saharan Africa.

## 1 INTRODUCTION

Indoor residual spraying (IRS) is a widely used public health intervention to reduce and ultimately interrupt malaria transmission by decreasing the lifespan of the female *Anopheles* mosquito (i.e., vector) (Bhatt et al., 2015; Pluess et al., 2010; World Health Organization, 2014, 2015b). This impact on transmission also leads to reduced incidence and prevalence of *Plasmodium* spp. infection in humans. To date there have not been any studies on the impact of IRS on parasite population genetics, yet it is reasonable to propose that reduced transmission and infection prevalence would lead to decreased *Plasmodium falciparum* diversity within individual human hosts and overall, in the population. Considering that sexual recombination (i.e., meiosis) is an obligatory part of the *P. falciparum* life cycle in the mosquito (Paul & Day, 1998), IRS should also lead to reduced outcrossing in the mosquito with consequences for the genetic structure of the parasite population.

Multilocus microsatellite genotyping is a validated tool for examining *P. falciparum* diversity and population structure and has been successfully used to characterise parasite populations across endemic regions in sub‐Saharan Africa, South America, Southeast Asia, and the Pacific (Anderson et al., 2000; Anthony et al., 2005; Barry et al., 2013; Kattenberg et al., 2020; Machado et al., 2004; Mobegi et al., 2012; Vera‐Arias et al., 2019; Yalcindag et al., 2012). These studies have shown that in high‐transmission settings (e.g., sub‐Saharan Africa) where the majority of infections are multiclonal, the *P. falciparum* population is characterised by high diversity, low levels of population differentiation, and linkage equilibrium, increasing the likelihood of recombination between genetically distinct parasite clones (i.e., outcrossing) in the mosquito following a blood meal. In contrast, low genetic diversity, extensive population differentiation, and strong linkage disequilibrium, are typically seen in low‐transmission regions (e.g., South America) or areas under intense control suggesting higher levels of inbreeding in these populations. The latter are “ideal elimination units” that may lead to a population bottleneck and where clonal parasites may be more readily lost to genetic drift events (Cotton et al., 2018; Escalante et al., 2015). Gene flow from higher transmission neighbouring regions can also increase diversity in areas of lower transmission or under malaria control (Branch et al., 2011; Roh et al., 2019). The high‐transmission genetic profile is characteristic of much of sub‐Saharan Africa that has been recently prioritized by the Roll Back Malaria Partnership and World Health Organization's (WHO) “High Burden to High Impact” (HBHI) country‐led approach to accelerate progress against malaria. Importantly the HBHI initiative points to IRS as one of the key interventions to enable high‐burden countries to get back on track towards malaria elimination (World Health Organization, 2019, 2020).

Malaria transmission remains highly seasonal across large parts of sub‐Saharan Africa, with peaks during the rainy season (i.e., high‐transmission season) and troughs in the dry season (i.e., low‐transmission season). Since mosquitoes require pools of water to breed, vector densities along with malaria transmission will increase during the wet season, while during the dry season fewer mosquitoes will propagate, leading to reduced transmission intensity. Previous studies have shown that in these areas characterized by high seasonal malaria transmission a large proportion of the human population across all ages harbour asymptomatic *P. falciparum* infections during both the wet and dry seasons (Galatas et al., 2016; Koram et al., 2003; Lindblade et al., 2013; Owusu‐Agyei et al., 2002; Tiedje et al., 2017). This reservoir of asymptomatic infections at the end of the dry season initiates transmission at the start of the next wet season and as such presents a target for interventions to potentially reduce or bottleneck the parasite population by vector control with IRS. The impact of this strategy was investigated by measuring microsatellite variation in the *P. falciparum* population in humans in an area characterized by high seasonal malaria transmission in Ghana, West Africa.

## 2 MATERIALS AND METHODS

### 2.1 Ethical approval

The study was reviewed and approved by the ethics committees at the Navrongo Health Research Centre, Ghana (NHRC IRB‐131), Noguchi Memorial Institute for Medical Research, Ghana (NMIMR‐IRB CPN 089/11‐12), The University of Melbourne, Australia (HREC 144‐1986 and HREC 195‐5652), and the University of Chicago, United States (IRB 14–1495). Individual informed consent was obtained in the local language from each enrolled participant by signature/thumbprint, accompanied by the signature of an independent witness. For children <18 years of age a parent or guardian provided consent. In addition, all children between the ages of 12 and 17 years provided assent.

### 2.2 Study area and design

**FIGURE 1:**
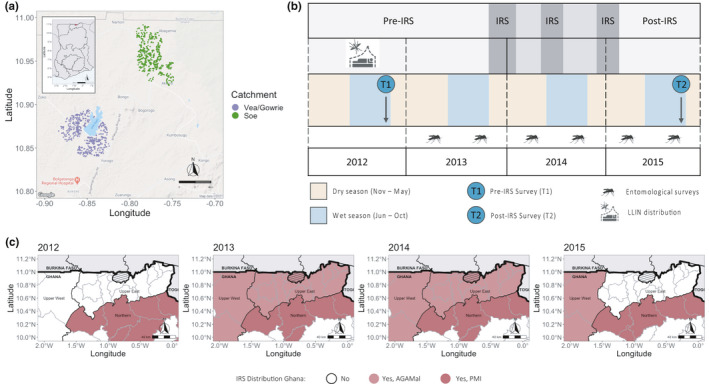
Study area, study design, and the rollout of indoor residual spraying (IRS) in northern Ghana. (a) The distribution of the compounds (i.e., households) included in this study from the two catchment areas in Bongo District: Vea/Gowrie, lower left (purple) and Soe, upper right (green). The compounds in Vea/Gowrie and Soe are approximately 20–40 km apart. The location of Bongo District in the Upper East Region of Ghana is shown in the insert map (upper left). Note: The human population in Bongo District resides in rural communities made up of small farm settlements scattered throughout the district. For the purposes of this study not all compounds in Bongo District were geolocated and therefore are not included on the map. (b) Study design showing the timing of the two age‐stratified cross‐sectional surveys (T1 and T2) in Bongo, Ghana. The first survey (T1) was conducted at the end of the wet season pre‐IRS in October 2012, while the second survey (T2) was conducted end of the wet season post‐IRS in October 2015. Three‐rounds of IRS with different organophosphates were implemented in the dry season across Bongo as indicated in grey: Round 1 (October 2013–January 2014, Vectoguard 40WP), Round 2 (May–July 2014, Actellic 50EC), and Round 3 (December 2014–February 2015, Actellic 300CS). Long‐lasting insecticidal nets (LLINs) were mass distributed in Bongo District by the NMCP/GHS between 2010–2012 as indicated. The mosquitos are used to denote the entomology surveys that were undertaken monthly between February 2013 and September 2015 in Bongo (see Supporting Information Methods). (c) Timing and distribution of IRS across the Upper East, Upper West, and Northern Regions of Ghana, West Africa between 2012 to 2015. Highlighted in dark red are the IRS programmes funded by the President's Malaria Initiative (PMI) and in light red are those funded by the Global Fund in partnership with the AngloGold Ashanti Malaria Control Programme (AGAMal). Bongo District is denoted with by the black hashed lines. The thick black lines are used to signify borders between Ghana and Burkina Faso (light grey) to the north, and Togo (light grey) to the west. Bongo District shares a northern border with the Nahouri Province in Burkina Faso where no IRS was implemented before and/or during the study period (PMI, 2017). Districts in Ghana where there is no shading (white) indicate that no IRS programmes were ongoing between 2012 and 2015

This study to investigate the impacts of an IRS intervention, under operational conditions, on the asymptomatic *Plasmodium falciparum* reservoir was conducted in Bongo District, located in the Upper East Region of Ghana (Figure 1a). Bongo District has a short but intense rainy season (~70 days with rain per year between June to October) and a prolonged dry season (November to May). Malaria in Bongo District is hyperendemic (i.e., consistent at high levels) and is characterised by marked seasonal transmission of *P. falciparum* (minor parasites: *P. malariae* and *P. ovale*) (Tiedje et al., 2017). For this study, participants were enrolled from two broad “catchment areas” (Vea/Gowrie and Soe) and hereinafter referred to collectively as “Bongo” for discussion purposes (Figure 1a). The catchment areas were considered to be different agroecological zones (irrigated vs. nonirrigated) based on their proximity to the Vea Dam, but were otherwise similar with respects to population size, age structure, and ethnic composition (Tiedje et al., 2017).

Using a cross‐sectional study‐design, two age‐stratified surveys of ~2000 participants per survey were undertaken at the end of the wet seasons (i.e., high‐transmission season) pre‐IRS (T1, October 2012) and post‐IRS (T2, October 2015) (Figure 1b). During each survey, relevant information was collected from all participants using structured questionnaires on their demographics, malaria history, and malaria prevention activities. Additional details on the study area, study population, sample collection procedures, etc. have been previously published (Tiedje et al., 2017). All individuals who were microscopically positive for *P. falciparum* (including mixed *P. falciparum*/*P. malariae* infections), were afebrile (axillary temperature <37.5°C) on the day the survey was conducted, and did not report a history of fever in the 24 h prior to being surveyed were defined as having an “asymptomatic *P. falciparum* infection” (hereafter designated as *P. falciparum* infections).

Over the last decade IRS has been scaled up across northern Ghana through ongoing support from the United States President's Malaria Initiative (PMI) and the Global Fund in partnership with the AngloGold Ashanti Malaria Control Programme (AGAMal) (Figure 1c) (National Malaria Control Programme, 2009, 2014; The Global Fund, 2012). Between 2013 and 2015, three‐rounds of IRS with different organophosphate formulations were implemented by AGAMal across all of the Upper East Region, including Bongo District, during the dry season before the high‐transmission wet season in attempts to interrupt transmission and reduce/bottleneck the parasite population (Figure 1c). Ideally IRS should be rolled out towards the end of the dry season to reduce transmission in the subsequent wet season. However, in Bongo District the timing of the three‐rounds of IRS varied due to logistical challenges, which are expected when implementing large‐scale interventions under operational conditions. For specific details on the monthly entomological surveys undertaken to monitor the impacts of IRS on the vector population in Bongo, please see Supporting Information Methods. Participant‐reported IRS coverage in Bongo ranged from 79.7% of compounds (i.e., households) in Round 1, to 96.6% and 96.1% in Rounds 2 and 3, respectively. The average coverage for all three‐rounds of IRS in Bongo was 90.8% of compounds, with no significant difference between the catchment areas surveyed in Bongo.

Several other malaria control interventions have been deployed in Ghana and Bongo District, including integrated vector‐control, management and treatment of uncomplicated malaria with artemisinin‐based combination therapies (ACTs), community mobilization and education, etc. (Ghana Health Service/Ministry of Health, 2009; National Malaria Control Programme, 2014). Long‐lasting insecticidal nets (LLINs) were mass distributed across Bongo District by the Ghana Health Service between 2010–2012 with support from UNICEF, Global Fund, and other partners (Smith Paintain et al., 2014; UNICEF, 2012; USAID Global Health Supply Chain Program, 2020). LLIN usage pre‐ and post‐IRS was high in Bongo, with 89.1% and 90.6% of participants, respectively, reporting sleeping under an LLIN the previous night (Table S1).

### 2.3 Microsatellite genotyping

For all participants with microscopically confirmed *P. falciparum* infections (i.e., isolates), two 5 x 5 mm sections were cut from each dried blood spot and placed in a 1.5‐ml centrifuge tube, with genomic DNA (gDNA) being extracted using the QIAmp DNA mini kit (Qiagen) as previously described (Tiedje et al., 2017). A subset of 200 microscopic *P. falciparum* isolates from both the pre‐IRS (T1) and post‐IRS (T2) surveys were selected for microsatellite genotyping based on their multiplicity of infection (MOI) (i.e., number of genetically distinct *P. falciparum* genomes) as determined using *var* genotyping (see Supporting Information Methods, Figure S1). Briefly, using this approach we estimated the MOI based on the number of *var* DBLα types identified per isolate, using a cutoff value of 60 *var* DBLα types per *P. falciparum* genome. Isolates with ≤60 *var* DBLα types were classified as single‐clone infections (MOI = 1), while isolates with >60 *var* DBLα types were classified as multiple‐clone infections (MOI > 1). To facilitate a more accurate assignment of the fluorescent peaks during the analysis (described below), only those isolates with a MOI = 1 or 2, were selected for the microsatellite genotyping (Anderson et al., 1999).

The *P. falciparum* isolates (*N* = 400) selected from the pre‐ and post‐IRS surveys were genotyped using a verified panel of 12 putatively neutral microsatellite markers located across the 14 chromosomes as described by Anderson et al. (1999): TA1, 2490, TA81, TA87, TA109, TA60, POLYA, TA42, ARA2, PfG377, PfPK2, and TA40, with modified cycling conditions as specified in Ruybal‐Pesántez et al. (2017). Fluorescently‐labelled PCR products were sent to a commercial sequencing facility (Macrogen Inc., South Korea) for capillary electrophoresis and fragment analysis on an Applied Biosystems 3730xl DNA analyser (ThermoFisher Scientific). Raw data files were imported using GeneMarker (SoftGenetics LLC), normalised based on the size standard LIZ500, and scored using customised panels as previously described (Anderson et al., 1999; Ruybal‐Pesántez et al., 2017). All major peaks that were within the expected marker base pair (bp) range and were spaced at intervals corresponding to trinucleotide (3 bp) repeats were considered to be true alleles. Any peak less than 33% of the primary peak (i.e., local max) for a locus was considered a minor allele and not interpreted as a true allele. Background noise was defined as any peak <200 fluorescent units (Anderson et al., 1999). These data were cleaned using R package base v. 3.5.0 (R Core Team, 2018) and then processed using TANDEM v. 1.09 (Matschiner & Salzburger, 2009), which is optimal to assign an allele to each trinucleotide microsatellite locus for each isolate. We combined data from the pre‐ and post‐IRS surveys prior to binning alleles with TANDEM to ensure each survey could be compared accurately to each other.

For the 200 isolates investigated in the pre‐ and post‐IRS surveys, the median genotyping success was 89.2% in the pre‐IRS survey and 92.8% in the post‐IRS survey for the 12 microsatellite markers (Table S2) as expected for low‐density asymptomatic *P. falciparum* infections. Since isolate genotyping success for TA1 and TA42 was <75% pre‐IRS and/or post‐IRS (Table S2), these loci were subsequently removed, with 10 microsatellite loci included for the downstream multilocus microsatellite analyses (Note: All 12 microsatellite loci, including TA1 and TA42, were successfully amplified and genotyped for the 3D7 positive controls, thus the possibility of null alleles could not be excluded). Finally, only those isolates with genotyping data at ≥3 microsatellite loci were included, resulting in 192 (96.0%) and 200 (100%) isolates from the pre‐ and post‐IRS surveys, respectively (Table 1, Figure S1).

Isolates with one peak at all microsatellite loci were defined as “true” single‐clone infections (MOI = 1). Isolates with two or more peaks at ≥1 loci were considered to be multiple‐clone infections (MOI > 1) and the multilocus haplotypes were constructed using the predominant peak at each locus. The combined data set with the single‐clone and multiple‐clone infections was defined as the “all infections” data set (Figure S1). Isolates with single‐clone infections and isolates with a maximum of two peaks at any locus (i.e., MOI = 2) were defined as the “dominant infections” data set (Ruybal‐Pesántez et al., 2017). The “dominant infections” data set is robust and accounts for possible confounding by multiple‐clone infections while still maximising the sample size available for analysis (Anderson et al., 1999).

### 2.4 Population genetic analyses

#### 2.4.1 Genetic diversity

Binned data files were processed manually in Microsoft Excel v. 16.30 to generate input files for the population genetics software packages, as described below. Patterns of genetic diversity were analysed using the R package *poppr v. 2.7.1* (Kamvar et al., 2014) which calculates the number of multilocus haplotypes (*h)*, the number of alleles per locus (*A*), the allele frequency per marker and expected heterozygosity (*H*_e_) between microsatellite marker pairs.

*H*_e_ was calculated using the formula:

$$H_{e}=\left(\frac{n}{n‐1}\right)1‐\sum_{i=1}^{k}p_{i}^{2}$$

Where *p* is the allele frequencies at a given locus and *n* is the number of observed alleles in each locus (Nei, 1978).

“Dominant” constructed haplotypes were used for these analyses. Allelic richness (*R*_s_) was calculated using the R package *PopGenReport v. 0.10* (Adamack & Gruber, 2014) to standardise and account for differences in sample sizes and genotyping success between surveys using a rarefaction method. Here, the sample size for each population and locus was set to the smallest number of alleles observed for a sample. The function Hs.test in the R package *adegenet v. 2.1.1* (Jombart, 2008) was used to test the difference in *H*_e_ between two populations (*x* and *y*): *H*_e_(*x*)−*H*_e_(*y*).

#### 2.4.2 Effective population size

The PGDSpider v 2.1.1.5 (Lischer & Excoffier, 2012) conversion tool for populations genetics and genomics programs was used to convert the data for BOTTLENECK v. 1.2.02 (Piry et al., 1999). BOTTLENECK was used to test if our populations experienced a bottleneck event, henceforth defined as a recent severe reduction in effective population size (*N*_e_). For selectively neutral loci, *A* and *H*_e_ result from an equilibrium between mutation and genetic drift (Luikart & Cornuet, 1998). In non‐bottlenecked populations that are near this “mutation‐drift” equilibrium, the *H*_e_ will equal the heterozygosity expected at mutation‐drift equilibrium (*H*_eq_) (i.e., *H*_e_ = *H*_eq_). If a bottleneck event has occurred, *A* decreases immediately but *H*_e_ is briefly retained, becoming larger than *H*_eq_ (i.e., *H_e_* > *H*_eq_) (Cotton et al., 2018; Luikart & Cornuet, 1998; Piry et al., 1999). This transient excess can be used to detect recent bottleneck events (within 2–4*N*_e_ generations), while population expansions typically exhibit a heterozygosity deficiency (where *H*_eq_ > *H*_e_) (Branch et al., 2011; Piry et al., 1999). *H*_eq_ is calculated from the observed *A* and the sample size of individuals. Note, the duration of complete parasite lifecycle from mosquito to mosquito is estimated to be six generations per year (Anderson et al., 2017; Hughes & Verra, 2001). Therefore, between the last round of IRS in February 2015 and the post‐IRS survey in October 2015, there would have been approximately 3–4 generations. This is a sufficient time period for the bottleneck calculation from Cornuet and Luikart (1996) to be able to predict whether there was a genetic bottleneck (Luikart & Cornuet, 1998).

Using 1000 simulations, the infinite alleles model (IAM) and stepwise mutation model (SMM) were run as recommended by BOTTLENECK and as reported in the literature (Anderson et al., 2000; Branch et al., 2011; Cornuet & Luikart, 1996; Jennison et al., 2015; Luikart & Cornuet, 1998; Piry et al., 1999). The IAM posits that every mutation event generates a new allele that is independent from its progenitor (Selkoe & Toonen, 2006), while the SMM contends that there is an equal probability that a mutation adds or subtracts one or more repeat units at a fixed rate. The SMM process mimics DNA replication errors that generate mutations and allows for a mutation to an existing allele (homoplasy) (Ellegren, 2004; Ohta & Kimura, 1973). This results in fewer distinct allele states than the IAM for the same mutation rate (Cornuet & Luikart, 1996).

For the SMM, *N*_e_ was calculated as:

$$N_{e}=\frac{\frac{1}{8}x\left\{{\left[\frac{1}{1‐H_{e}}\right]}^{2}‐1\right\}}{\mu }$$

And for the IAM, *N*_e_ was calculated by:

$$N_{e}=\left(\frac{H_{e}}{1‐H_{e}}\right)x\frac{1}{\mu }$$

Where *H*_e_ is the mean expected heterozygosity across all loci and *µ* is the microsatellite mutation rate for *P. falciparum*: 1.59 × 10^−4^ [95% confidence interval, 6.98 × 10^−5^–3.7 × 10^−4^] (Anderson et al., 2000).

The SMM has been identified as a more stringent model for microsatellite data and was ultimately used for analysis (Cornuet & Luikart, 1996; Luikart & Cornuet, 1998; Piry et al., 1999). A one‐tailed Wilcoxon's sign rank test was used to detect heterozygosity excess using allele frequency data (Luikart & Cornuet, 1998). Furthermore, BOTTLENECK was used to compare the distribution of allele frequencies observed in a population to the distribution expected in a nonbottlenecked population (Luikart et al., 1998). Nonbottlenecked populations would have a large proportion of alleles at low‐frequency resulting in an L‐shaped distribution, while bottlenecked populations have a shifted mode of distribution where low‐frequency alleles become less abundant (Luikart et al., 1998).

#### 2.4.3 Multilocus linkage disequilibrium

The extent of inbreeding within and between populations was estimated using the R package *poppr v. 2.7.1* (Kamvar et al., 2014). The standardised index of association (${\bar{r}}_{d}$) (Agapow & Burt, 2001) was used to estimate the extent of multilocus linkage disequilibrium (LD, i.e., the nonrandom association of alleles) and is based on the index of association (*I*_A_) (Smith et al., 1993). *I*_A_ has been shown to increase steadily with the number of loci, therefore the standardised form, ${\bar{r}}_{d}$, was used to account for the number of loci sampled (i.e., 10 microsatellite loci) to reduce bias. To uncover whether any patterns of LD were due to a single pair of loci, or if there were any significantly associated pairs of loci masked by an insignificant overall LD, we calculated the ${\bar{r}}_{d}$ for all microsatellite pairs (pairwise ${\bar{r}}_{d}$). The ${\bar{r}}_{d}$ and pairwise ${\bar{r}}_{d}$ among loci were estimated using a Monte Carlo simulation method of 9999 samplings, where alleles were reshuffled at random among haplotypes. This analysis tested our ${\bar{r}}_{d}$ values against the null distribution of no linkage among/between markers, as expected for a randomly mating population (Agapow & Burt, 2001; Kamvar et al., 2014). To calculate ${\bar{r}}_{d}$ we only used those isolates in the “dominant infections” data set that had complete infection haplotypes (i.e., no missing data) to ensure that the permutation analysis shuffled the alleles per haplotype without bias.

#### 2.4.4 Genetic relatedness

To investigate genetic relatedness between isolates we calculated the pairwise allele sharing (*P*_AS_) statistic using only those isolates in the “dominant infections” data set that had complete infection haplotypes. Complete multilocus haplotypes were used to ensure that the denominator would be consistent for all comparisons.

*P*_AS_ scores were calculated by:

$$P_{\text{AS}}=\frac{N_{\text{AB}}}{N_{L}}$$

Where *N*_AB_ is the number of alleles shared between two infection haplotypes and *N*_L_ is the microsatellite number of microsatellite loci (i.e., 10) (Ruybal‐Pesántez et al., 2017).

The resulting *P*_AS_ score is represented as a ratio, ranging from 1 (i.e., clones) to 0 (i.e., unrelated). Any infection haplotype pairs with a *P*_AS_ ≤ 0.25 would be considered “unrelated”, 0.25 < *P*_AS_ < 0.5 would be considered “half‐siblings”, and a *P*_AS_ ≥ 0.5 would be considered “related” (i.e., siblings or recent recombinants). *P*_AS_ comparisons were calculated between all possible infection haplotype pairs both within (i.e., pre‐ and post‐IRS) and between (i.e., pre‐ vs. post‐IRS) the survey time points investigated.

To visualise whether the “related” infection haplotypes were clustered geographically, we constructed spatial genetic relatedness networks at a threshold of *P*_AS_ ≥ 0.70 (i.e., identical at ≥7 of the 10 microsatellite loci). This threshold was selected to visualise the genetic similarity between isolates that probably resulted from a recent transmission and/or recombination event. These spatial networks were plotted using the R packages *ggraph v. 1.0.2* (Pedersen, 2020), *tidygraph v. 1.1.2* (Pedersen, 2019) and *ggmap v. 3.0.0.90* (Kahle & Wickham, 2013) using each isolate's compound/household GIS coordinates in Bongo for mapping.

#### 2.4.5 Genetic differentiation

To measure the degree of genetic differentiation of *P. falciparum* populations between timepoints, catchment areas, and villages, *G_ST_* and Jost's differentiation indices (*D*) were calculated using the R package *DEMEtics v. 0.8‐7* (Gerlach et al., 2010). Jost's *D* is a heterozygosity‐based estimator of population differentiation and the evolution of genetic divergence between populations (Gerlach et al., 2010; Jost et al., 2018). This measure is robust when the number of observed alleles per locus is greater than two and when the allelic diversity for a locus (within‐population diversity) is high (Gerlach et al., 2010). *G*_ST_ measures the extent of fixation of alleles per population, while Jost's *D* measures the extent of private alleles in a population and provides a more accurate measure of genetic differentiation between populations (Jost et al., 2018). For more details on these measures of genetic differentiation refer to the Supporting Information Methods.

#### 2.4.6 Population structure

Parasite population genetic structure was further investigated using the Bayesian clustering software STRUCTURE v. 2.3.4 (Pritchard et al., 2000). This program uses a Bayesian model to cluster haplotypes into distinct genetic populations (*K*) based on their inferred ancestry as determined by allele frequencies at each locus (Pritchard et al., 2000). Haplotypes from both the pre‐ and post‐IRS surveys were input together and the simulations were run stratified at the spatial level by Bongo and by catchment area (i.e., Vea/Gowrie and Soe). The analyses were run for *K* = 1 to 10 (*n* catchments +2) with 20 stochastic simulations for each *K* and 100,000 Markov chain Monte Carlo iterations, after a burnin period of 100,000 using the admixture model and assuming correlated allele frequencies. The STRUCTURE program output contains the log probability of the data, LnP[*D*], which can be used to determine the optimal *K* cluster (Pritchard et al., 2000). A second order rate of change of LnP[*D*], Δ*K*, was calculated according Evanno et al. (2005), which has been found to be a more sensitive method of predicting the real number of clusters. STRUCTURE Harvester v. 0.6.94 (Earl & vonHoldt, 2012) was used to process the results and calculate the optimal number of clusters from the peak Δ*K* according to the Evanno et al. (2005) method. CLUMPAK (Clustering Markov Packager Across *K*) v. 1.1 (Kopelman et al., 2015) was used to account for the number of stochastic simulations per *K* and to visualise the results at using time and spatial levels.

#### 2.4.7 Malaria epidemiology statistical analyses

R v. 3.5.0 (R Core Team, 2018) implemented in RStudio v.1.1.383 (RStudio Team, 2015) was used for the statistical analyses. For each survey, study participants were categorised into defined age groups (1–5, 6–10, 11–20, 21–39, and ≥40 years), sex, catchment areas (Vea/Gowrie and Soe), LLIN usage the previous night, and antimalarial use in the two weeks prior to being surveyed, as described in the study design. Continuous variables are presented as medians with interquartile ranges (IQRs) and discrete variables are presented using the calculated/observed prevalence values with 95% confidence intervals (CIs). Fisher's exact or chi‐squared tests (*X* ^2^) were used for univariate analyses of discrete variables to compare proportions; nonparametric Wilcoxon signed rank test (comparing distributions across two paired groups), Mann–Whitney *U* (comparing distributions across two groups), Kruskal–Wallis (comparing distributions across *k* groups) and tests were used to compare distributions for continuous variables. A test was deemed statistically significant if the *p*‐value was <.05.

## 3 RESULTS

### 3.1 Parasitology changes after IRS

IRS was deployed three times across Bongo District between 2013 and 2015 during the dry season to reduce the size of the parasite population that initiates malaria transmission in the subsequent wet season. By comparing the *Plasmodium falciparum* parasite populations at the end of the wet season pre‐ to post‐IRS, we tested the hypothesis that by interrupting transmission, IRS could lead to a population genetic bottleneck. Following three‐rounds of IRS, the prevalence of *P. falciparum* infections across all ages in Bongo decreased significantly from 42.0% in the pre‐IRS survey (T1, October 2012) to 27.0% in the post‐IRS survey (T2, October 2015) (i.e., 35.7% reduction, *p* > .001, Table S3). During both the pre‐ and post‐IRS surveys the highest prevalence of infection occurred among the older children (6–10 years) and adolescent (11–20 years) age groups, with prevalence decreasing as expected for those >20 years. There were significant reductions in *P. falciparum* prevalence by age between the pre‐ to post‐IRS surveys, specifically among the youngest children (1–5 years; 48.6% (pre‐IRS) to 15.6% (post‐IRS)) and the oldest children (6–10 years; 61.5% (pre‐IRS) to 40.8% (T2)) surveyed (*p* < .001) (Table S3). During the pre‐IRS survey, there were spatial differences between the catchment areas, with the prevalence of infection in Soe (45.0%) being significantly greater in comparison to Vea/Gowrie (35.5%) (*p* = .005) (Table S3). However, post‐IRS there were no significant differences in *P. falciparum* prevalence between these catchment areas. In addition to prevalence, median *Plasmodium* spp. density significantly decreased between the pre‐IRS (520 parasites/µl) and post‐IRS (320 parasites/µl) surveys (*p* < .001) (Table S3). These significant reductions in parasitemia were observed after stratifying by both sex and catchment area (*p* ≤ .006). It should however be noted that for all age groups surveyed, except those ≥40 years (*p* < .001), there were no significant changes in parasitemia between the pre‐ and post‐IRS surveys. Finally, median MOI (defined using *var* genotyping, see Supporting Information Methods) significantly decreased following the IRS intervention from a MOI = 3 [95% CI: 1–4] pre‐IRS to a MOI = 1 [95% CI: 1–2] post‐IRS (*p* < .001) (Table S3). These significant changes in MOI were observed across all age groups, sexes, and catchment areas.

### 3.2 Microsatellite study population

Using the 10 microsatellites markers, a subset of 192 and 200 participants with microscopic *P. falciparum* infections from the pre‐ and post‐IRS surveys, respectively, were used for the microsatellite genotyping (Table 1). This subset of isolates selected based on MOI (see Section 2) was not statistically different than those isolates excluded for any of the key variables *(p* > .05) (Table S4), except for age pre‐IRS (χ^2^ = 15.46, *p* < .001) and parasitaemia post‐IRS (*p* < .01). For the subset of participants selected, there were no significant differences between the pre‐ and post‐IRS surveys for any of the demographic or parasitological parameters, except for age and reported antimalarial usage (*p* ≤ .002, Table 1). These age‐specific differences were in the youngest (1–5 years) and oldest (6–10 years) children's age groups, such that there were fewer 1–5 year olds included post‐IRS compared to pre‐IRS (8.5% vs. 18.8% respectively) and a greater number of 6–10 year olds included post‐IRS compared to pre‐IRS (34.0% vs. 19.3%, respectively) (Table 1). These proportional differences, although significant, reflect the underlying epidemiological changes in the prevalence of *P. falciparum* infections by age following the IRS intervention with fewer 1–5 year olds being infected post‐IRS (Tables S3 and S4). For antimalarial drug usage in the previous two weeks, similar to the patterns seen in the Bongo study population (Table S1), we found that reported antimalarial usage significantly declined following the IRS intervention (43.2% vs. 11.5%, pre‐ vs. post‐IRS, respectively, *p* < .001) (Table 1). The complete demographic and parasitological characteristics of the 192 and 200 *P. falciparum* isolates analysed by multilocus microsatellite genotyping in the pre‐ and post‐IRS surveys, are presented in Table 1.

**TABLE 1:** Demographics and parasitological characteristics of the participants analysed with *P. falciparum* infections (including mixed *P. falciparum*/*P. malariae* infections) for the pre‐IRS (T1, October 2012) and post‐IRS (T2, October 2015) surveys

Characteristic	Pre‐IRS (T1, October 2012)	Post‐IRS (T2, October 2015)	*p*‐value
Total population	192	200	
Age groups^a^			
1–5 years	36 (18.8)	17 (8.5)	.002
6–10 years	37 (19.3)	68 (34.0)	
11–20 years	52 (27.1)	58 (29.0)	
21–39 years	27 (14.1)	20 (10.0)	
≥40 years	40 (20.8)	37 (18.5)	
Sex^a^			
Female	101 (52.6)	98 (49.0)	.476
Male	91 (47.4)	102 (51.0)	
Catchment area^a^			
Vea/Gowrie	87 (45.3)	103 (51.5)	.220
Soe	105 (54.7)	97 (48.5)	
Reported LLIN usage^b^			
Yes	163 (84.9)	180 (90.0)	.127
No	29 (15.1)	20 (10.0)	
Reported antimalarial usage^c^			
Yes	83 (43.2)	23 (11.5)	<.001
No	109 (56.8)	177 (88.5)	
*Plasmodium* spp. median density^d^			
All	240 [120–2020]	320 [120–1190]	.565
Age groups			
1–5 years	6480 [230–46,620]	1640 [320–17,280]	<.001
6–10 years	360 [120–1480]	360 [160–1430]	
11–20 years	160 [80–610]	340 [160–1060]	
21–39 years	160 [80–740]	320 [120–2200]	
≥40 years	220 [110–1150]	120 [80–480]	
Sex			
Female	240 [120–1560]	320 [120–1520]	.555
Male	240 [120–2940]	360 [160–1080]	
Catchment area			
Vea/Gowrie	200 [120–1140]	360 [160–1120]	.783
Soe	400 [120–2400]	320 [120–1280]	
*P. falciparum* median MOI^e^			
All	1 [1–2]	1 [1–2]	.104
Age groups			
1–5 years	2 [1–2]	1 [1–2]	.002
6–10 years	2 [1–2]	1 [1–2]	
11–20 years	1 [1–2]	1 [1–2]	
21–39 years	1 [1–2]	1 [1–1]	
≥40 years	1 [1–2]	1 [1–2]	
Sex			
Female	1 [1–2]	1 [1–2]	.343
Male	1 [1–2]	1 [1–2]	
Catchment area			
Vea/Gowrie	1 [1–2]	1 [1–2]	.003
Soe	2 [1–2]	1 [1–2]	

Abbreviations: IQR, interquartile range; LLIN, long‐lasting insecticidal nets; MOI, multiplicity of infection.

^a^ Data reflect No. (% [*n*/*N*]) of participants sampled that were positive for *P. falciparum* (including mixed *P. falciparum*/*P. malariae* infections) by microscopy.

^b^ Participant self‐reported LLIN usage the previous night

^c^ Participant self‐reported antimalarial treatment in the two weeks prior to being surveyed.

^d^ Median parasite density for the microscopically positive *P. falciparum* (including mixed *P. falciparum*/*P. malariae* infections) (value/µl [IQR]) isolates.

^e^ Data reflect the median estimated MOI based on *var* genotyping (see Supporting Information Methods).

### 3.3 Microsatellite genetic diversity of *P. falciparum*

**FIGURE 2:**
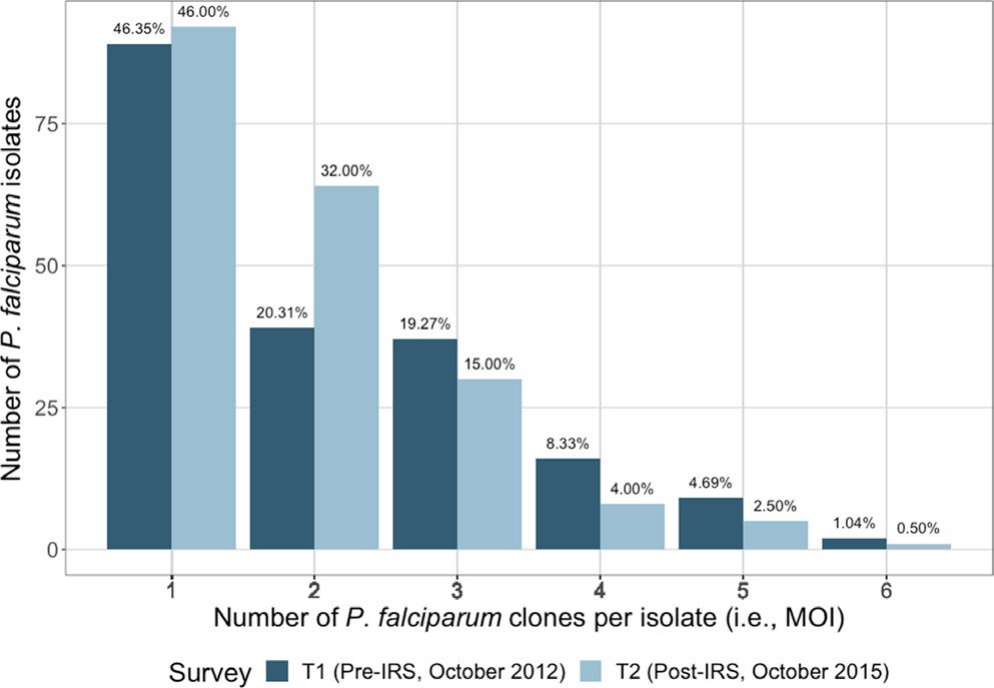
Distribution of the number of *P. falciparum* clones (i.e., multiplicity of infection (MOI)) in each *P. falciparum* isolate sampled pre‐IRS (T1, *N* = 192, dark blue) and post‐IRS (T2, *N* = 200, light blue) based on the microsatellite genotyping. There were no significant differences in the proportion of clones from pre‐ to post‐IRS (χ^2^ = 10.833, *p* = .055). Note: The numbers reflect the percentage of participants pre‐ and post‐IRS (%, *n*/*N*) in each MOI category

Based on microsatellite genotyping, 53.6% and 54.0% of the isolates in the pre‐ and post‐IRS surveys, respectively, had an MOI > 1, indicating the majority of population in Bongo harboured multiple‐clone infections pre‐ and post‐IRS (Figure 2). In Bongo, high levels of genetic diversity were observed pre‐ and post‐IRS for both the “all infections” and “dominant infections” data sets (Table S5 and Table 2) . Since measures of diversity were comparable between these data sets, the “dominant infections” data set was used for all the subsequent analyses to maximise the sample size available for analysis while minimizing the possible confounding effects of including multiclonal infections (i.e., “all infections” data set) (see Section 2; Tables S6–S8). The dominant infections” data set includes 128 and 156 isolates pre‐ and post‐IRS, respectively (Table 2, Figure S1).

**TABLE 2:** Patterns of *P. falciparum* genetic diversity using the “dominant infections” data set for the pre‐IRS (T1, October 2012) and post‐IRS (T2, October 2015) surveys (see Tables S5 and S9 for additional information)

Population	*n*		*h*		*A*			*R*_s_			*H*_e_		
	T1	T2	T1	T2	T1	T2	*p*‐value^†^	T1	T2	*p*‐value^†^	T1	T2	*p*‐value^‡^
Vea/Gowrie	56	80	56	79	9.7	10.9	.854	9.2	9.3	.991	0.79	0.81	.105
Soe	72	76	72	76	10.0	10.4	.950	8.9	9.1	.968	0.79	0.80	.396
Total	128	156	128	155	11.5	12.4	.897	11.4	11.6	.968	0.79	0.81	.048*

*p*‐value = T1 and T2 comparison. *p‐value* ^†^ calculated by chi‐square test; *p‐value* ^‡^ calculated by “Hs.test” function. *p‐value < .05.

Abbreviations: *A*, mean number of alleles per locus; *H* *_e_*, expected heterozygosity; *h*, number of haplotypes; n, number of isolates; *R* *_s_*, allelic richness estimate.

**FIGURE 3:**
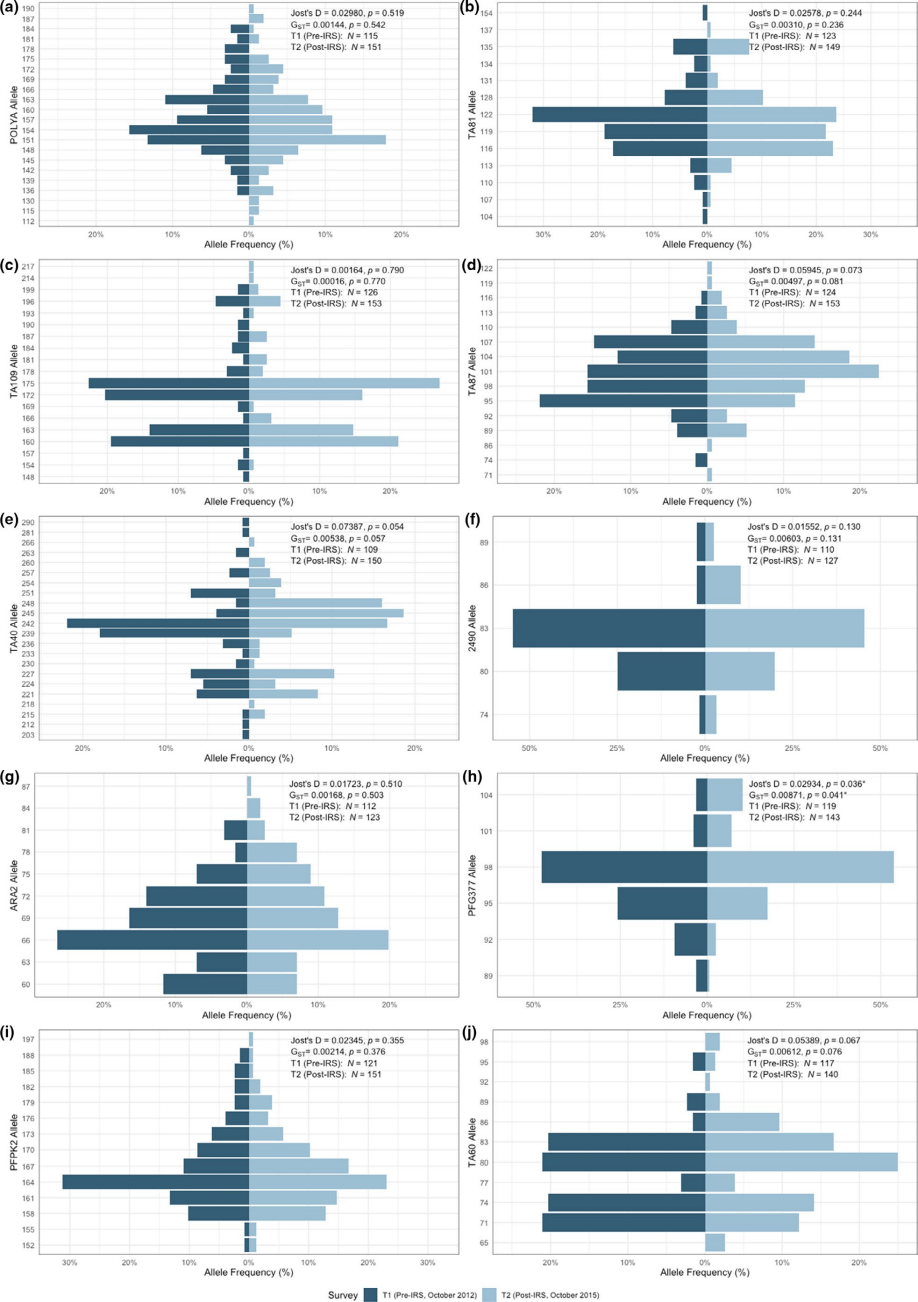
Distribution of allele frequencies for the microsatellite loci genotyped pre‐IRS (T1, October 2012) and post‐IRS (T2, October 2015) using the “dominant infections” data set. The Jost's *D* and *G_ST_* values have been provided for each locus, along with the number of isolates that were genotyped per locus. For the number of isolates (*N*) with data for each locus, please see Table S6 for more details

All the microsatellite loci genotyped were polymorphic and ranged from five to 20 alleles per locus pre‐IRS and five to 22 alleles post‐IRS (Tables S6 and S7). When the allele frequency distributions were compared between the pre‐ and post‐IRS surveys, four loci varied significantly: TA87 (*p* = .050), ARA2 (*p* = .040), PFG377 (*p* < .001) and TA60 (*p* = .030) (Figure 3). Alleles observed only at one time point (i.e., pre‐ or post‐IRS) were defined as “private alleles” and there were more private alleles observed post‐IRS compared to pre‐IRS, mostly at a low frequency for six loci (Figure 3, Tables S6‐S8).

In both the pre‐ and post‐IRS surveys there was considerable allelic variation defined using both the number of alleles per locus (*A*) and allelic richness estimates (*R*_s_) (Table 2). There was a mean of 11.5 and 12.4 alleles per locus pre‐ and post‐IRS, respectively, and a comparable estimated *R*_s_ of 11.4 pre‐IRS and 11.6 post‐IRS (Table 2). We also observed similar trends in *A* and *R*_s_ for both catchment areas during the pre‐ and post‐IRS surveys (Table 2). In addition, despite the observed reduction in transmission intensity following the IRS intervention (as measured by the entomological inoculation rate (EIR), see Supporting Information Methods) there were no significant changes in the population genetic measures of allelic variation described above (Table 2). We also observed similar trends in *A* and *R*_s_ for both catchment areas during the pre‐ and post‐IRS surveys, as well as no significant changes in these parameters pre‐ to post‐IRS (Table 2).

The same number of haplotypes as isolates was observed pre‐IRS, thus every multilocus haplotype was unique (Table 2). Post‐IRS every haplotype was also unique, except for two individuals from the same compound (i.e., household) in Vea/Gowrie that shared the same infection haplotype (Table 2). In Bongo, *H*_e_ remained high post‐IRS despite the IRS intervention with a significant, but slight increase in *H*_e_ between in the pre‐ and post‐IRS surveys (pre‐IRS = 0.79 vs. post‐IRS = 0.81, *p* = .048, Table 2, Tables S5 and S9). This significant difference however was not maintained when we stratified by catchment area, indicating potential temporal differences.

### 3.4 *N*_e_ estimates and population bottleneck investigation

Effective population size (*N*_e_) was calculated to estimate the seeding parasite populations in Bongo. *N*_e_ increased pre‐ to post‐IRS for both the stepwise mutation model (SMM) and infinite alleles model (IAM) (Table 3, Tables S10 and S11). To quantify whether there was a recent bottleneck event for the time point comparisons (i.e., pre‐ and post‐IRS surveys), we calculated the number of loci in heterozygosity excess or deficiency. A transient excess of heterozygosity would be indicative of a recent genetic bottleneck (see Section 2). More loci exhibited a heterozygosity deficiency (i.e., *H*_eq_ > *H*_e_) when comparing the pre‐ and post‐IRS surveys under the more conservative SMM, indicating that the parasite populations in Bongo had not undergone a bottleneck at either time point comparison (Table 4; Tables S10 and S11). This is consistent with our findings above where the number of alleles (*A*), and therefore *H*_e_, was maintained at the levels observed before the introduction of the IRS intervention in Bongo. This suggests that there was no bottleneck event between the pre‐ and post‐IRS surveys investigated (i.e., T1 and T2).

**TABLE 3:** Effective population size (*N*_e_) estimates for the *P. falciparum* populations using the stepwise mutation model (SMM) and the infinite alleles model (IAM) in the pre‐IRS (T1, October 2012) and post‐IRS (T2, October 2015) surveys

Time point	SMM	IAM
Pre‐IRS (T1, October 2012)	16,131 [6932–36,746]	5721 [2459–13,033]
Post‐IRS (T2, October 2015)	19,196 [8249–43, 727]	6355 [2731–14,745]

Note

*N*_e_ estimates are based on mean effective heterozygosity and estimated *P. falciparum* microsatellite mutation rate 1.59 × 10^−4^ [95% confidence interval: 6.98 × 10^−5^–3.7 × 10^−4^] (Anderson et al., 2000). The upper and lower confidence intervals for the mutation rate used to estimate *N*_e_ are labelled in parentheses.

Abbreviations: IAM, infinite alleles model; SMM, stepwise mutation model.

**TABLE 4:** Number of loci with excess or deficiency in the heterozygosity (*H*_e_) relative to the heterozygosity at mutation‐drift equilibrium (*H*_eq_) for the *P. falciparum* populations in the pre‐IRS (T1, October 2012) and post‐IRS (T2, October 2015) surveys

Population	*H*_e_ excess	*H*_e_ deficiency	*p*‐value	Mode‐shift
Pre‐IRS (T1, October 2012)	4	6	.984	Normal
Post‐IRS (T2, October 2015)	1	9	.999	Normal

Note

Normal: normal L‐shaped distribution = nonbottlenecked population, Shifted: shifted mode = bottlenecked population. *H*_e_ excess is the transient increase in the *H*_eq_ compared to *H*_e_ observed for the population, while *H*_e_ deficiency is a lower *H*_eq_ than *H*_e_ observed.

Data were subject to mutation drift equilibrium via SMM and mode‐shift analyses (BOTTLENECK v. 1.2.02). *p*‐value tests an excess of *H*_e_ via a Wilcoxon signed‐rank test.

Abbreviations: *H*_e_, heterozygosity; *H*_eq_, heterozygosity at mutation‐drift equilibrium.

### 3.5 Multilocus linkage disequilibrium

**FIGURE 4:**
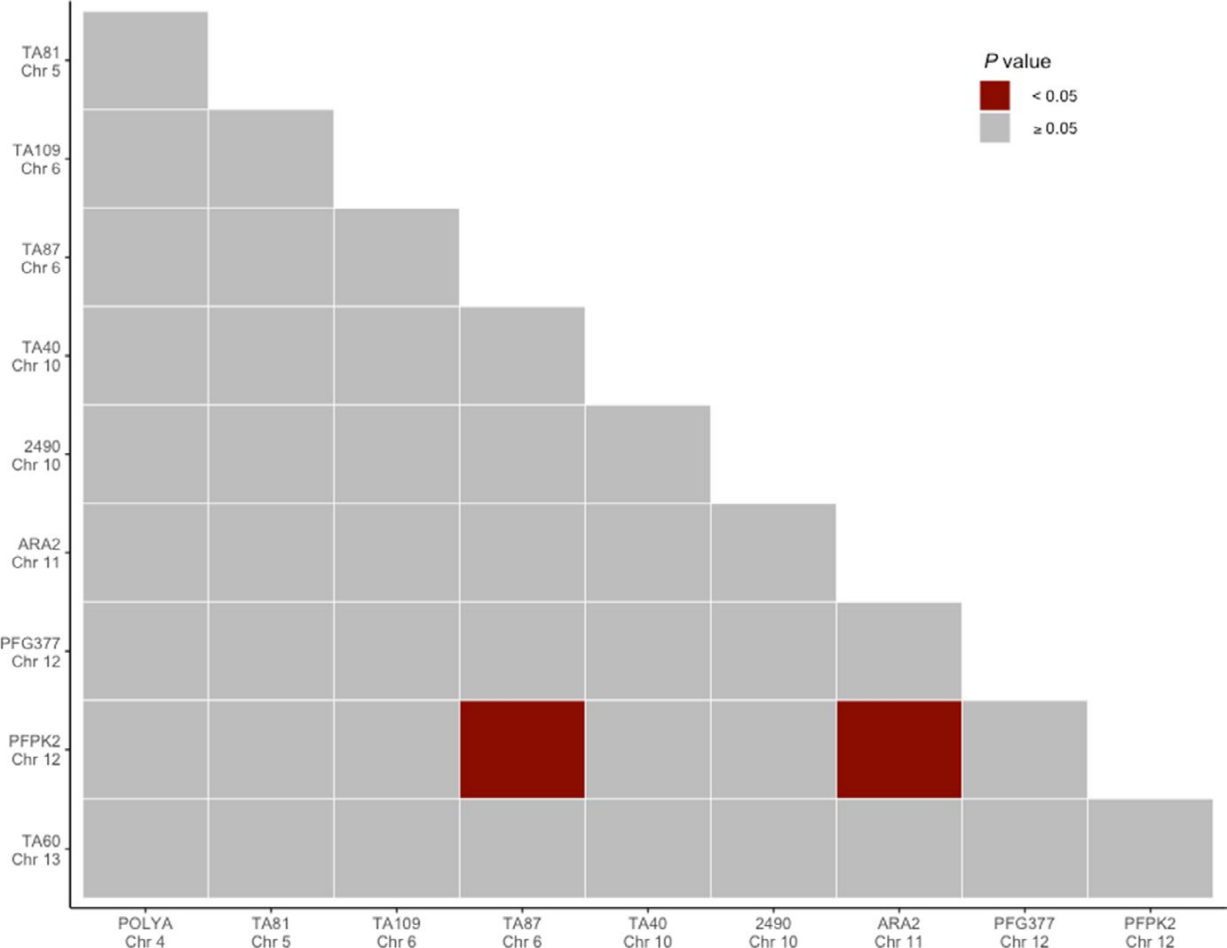
Pairwise linkage disequilibrium (${\bar{r}}_{d}$) for the *P. falciparum* infections using the “dominant infections” with complete haplotypes (i.e., no missing data, see Section 2) pre‐IRS (T1, October 2012) and post‐IRS (T2, October 2015) (*N* = 165). The colour key provided corresponds to the *p*‐value for each pairwise comparison where grey indicates a nonsignificant *p*‐value (*p *≥ .05) and red represents a significant *p*‐value (*p* < .05)

The index of association, ${\bar{r}}_{d}$, was used to assess multilocus linkage disequilibrium (LD), or nonrandom associations among loci using only the “dominant infections” with complete haplotypes (*N* = 81 pre‐IRS and *N* = 84 post‐IRS, (see Section 2 and Figure S1). Given that we examined putatively neutral loci across the genome, significant multilocus LD may provide evidence of past and/or current selection on the local parasite population (e.g., antimalarial drug selection (Ruybal‐Pesántez et al., (2017))). Using this analysis, we did not detect any significant multilocus LD in Bongo or in either catchment area, within (i.e., pre‐ and post‐IRS) and between (i.e., pre‐ vs. post‐IRS) the time point surveys (Table 5). To investigate whether this insignificant multilocus LD might actually be masking LD between specific pairs of loci (Ruybal‐Pesántez et al. (2017), we next examined the locus‐by‐locus pairwise ${\bar{r}}_{d}$ (Figure 4). Using this approach, we observed that although specific pairs of loci were in LD, there was no evidence of physical linkage as none of the loci pairs identified were on located on the same chromosome (Figure 4, Figure S2). These results are expected in an area with a high rate of sexual recombination/outcrossing.

**TABLE 5:** Multilocus linkage disequilibrium calculated using the "dominant infections" with complete haplotypes in in the pre‐IRS (T1, October 2012) and post‐IRS (T2, October 2015) surveys

Population	*n*	${\bar{r}}_{d}$ (*p*‐value)
Pre‐IRS (T1, October 2012)	81	0.00262 (.231)
Vea/Gowrie	28	0.01440 (.066)
Soe	53	–0.00247 (.673)
Post‐IRS (T1, October 2015)	84	0.00446 (.114)
Vea/Gowrie	48	0.01230 (.019)
Soe	36	0.00727 (.163)
Total	165	0.00171 (.221)

Note

Abbreviations: *n,* Number of isolates; ${\bar{r}}_{d}$ standardised index of association by Agapow and Burt (2001).

*p*‐values for each population obtained from 9999 permutations of the data are labelled in brackets.

### 3.6 Genetic relatedness of *P. falciparum* infection multilocus haplotypes

**FIGURE 5:**
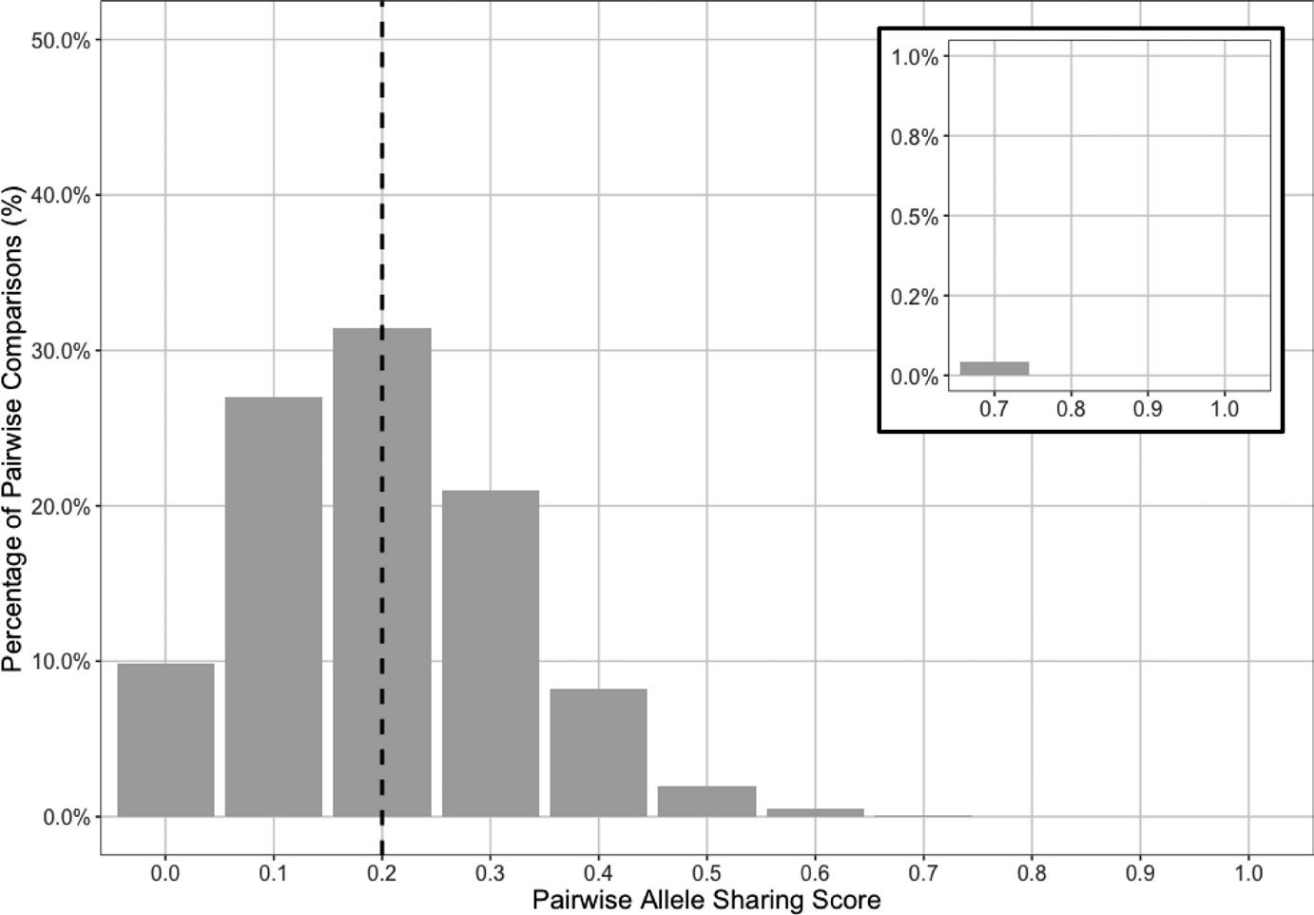
Distribution of the pairwise allele sharing (*P*_AS_) scores overtime by comparing the "dominant infections" with complete haplotypes (i.e., no missing data, see Section 2) from the pre‐IRS survey (T1, October 2012) to those in the post‐IRS survey (T2, October 2015). The median *P*_AS_ was 0.2 and is indicated by the black dotted line. The *P*_AS_ scores between 0.7 to 1.0 are shown in the upper right insert. There were 6804 pairwise comparisons between the 81 haplotypes from the pre‐IRS survey (T1) and the 84 haplotypes from the post‐IRS survey (T2) compared (see Table S12)

Pairwise allele sharing (*P*_AS_) comparisons between infection haplotypes were next used to examine parasite genetic relatedness. Even though all the multilocus haplotypes were unique (i.e., not repeated, except for one pair as discussed), it is possible that these infection haplotypes may only differ at one or two loci and may reflect parasites that are highly related and/or have recently undergone sexual recombination in the mosquito (i.e., outcrossing).Using the dominant infections with complete haplotypes, we calculated *P*_AS_ scores both within (i.e., pre‐ and post‐IRS) and between (i.e., pre‐ vs. post‐IRS) the time point surveys. Using this analysis, we found that both pre‐ and post‐IRS the majority of infection haplotypes were unrelated and only shared ≤0.2 of their alleles (i.e., identical at two or fewer loci out of 10) (Figure S3, Table S12). When the infection haplotypes were compared over time between the pre‐ and post‐IRS surveys, they were all highly unrelated (median *P*_AS_ = 0.2), indicating that parasite clones (as defined by their multilocus microsatellite haplotypes) did not appear to be maintained temporally in Bongo (Figure 5, Table S12). In fact, of the 6,804 pairwise comparisons between (i.e., pre‐ vs. post‐IRS) the time point surveys, only 172 isolate pairs (2.5%) were “related” (i.e., siblings or recent recombinants) having haplotypes that shared ≥5 loci out of 10 loci (i.e., *P*_AS_ ≥ 0.5), and just three isolate pairs (0.04%) were “highly related” being identical at ≥7 out of 10 loci (i.e., *P*_AS_ ≥ 0.7) (Figure 5, Table S12). Multilocus haplotype pairs were significantly less related post‐IRS than pre‐IRS (Wilcoxon test, *p* < .001, Table 6), suggesting that there was a reduction in sexual recombination/outcrossing following the IRS intervention.

**TABLE 6:** Comparing the distribution of pairwise allele sharing (*P*_AS_) scores between the "dominant infections" with complete haplotypes in the pre‐IRS (T1, October 2012) and post‐IRS (T2, October 2015) surveys in each catchment area and Bongo

Population	Pre‐IRS (T1) vs. Post‐IRS (T2)	
	*n*	*p*‐value
Vea/Gowrie	76	.752
Soe	89	<.01
Bongo	165	<.001

*p*‐value was calculated by Wilcoxon‐rank test, comparing the distribution of *P*_AS_ scores over time.

^a^ Vea/Gowrie (T1 vs. T2) contains 1344 pairwise comparisons of 28 haplotypes from T1 and 48 haplotypes from T2. For Soe (T1 vs. T2) contains 1908 pairwise comparisons of 53 haplotypes from T1 and 36 haplotypes from T2. For Bongo (T1 vs. T2) contains 6804 pairwise comparisons of 81 haplotypes from T1 and 84 haplotypes from T2.

### 3.7 Geographic population structure between *P*. *falciparum* populations

#### 3.7.1 Pairwise allele sharing networks

**FIGURE 6:**
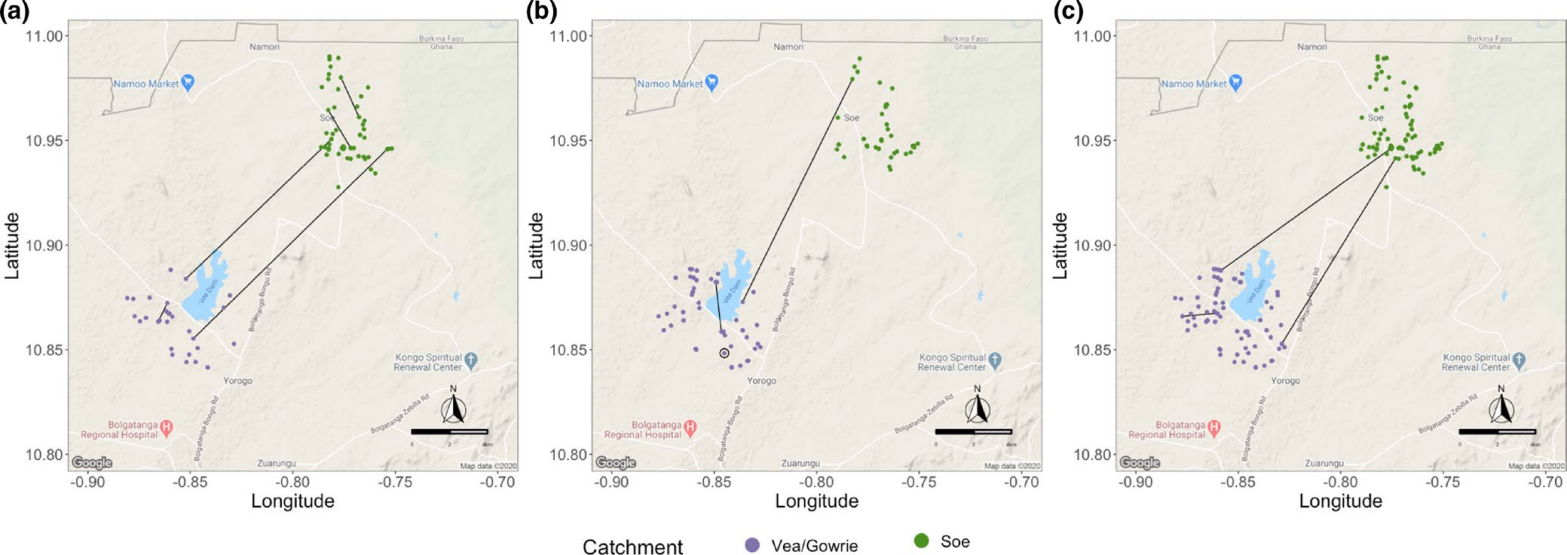
The genetic relatedness networks visualized spatially in Bongo for (a) pre‐IRS (T1, October 2012), (b)post‐IRS (T2, October 2015), and (c) pre‐ versus post‐IRS. These networks were constructed using the "dominant infections" with complete haplotypes pre‐IRS (*N* = 81 isolates compared), post‐IRS (*N* = 84 isolates compared), and pre‐ versus post‐IRS (*N* = 165 isolates compared). Each node represents an isolate and its geographic location in Bongo (i.e., compound/household location in each catchment area): purple corresponds to isolates in Vea/Gowrie and green corresponds to isolates in Soe. The edges in the networks (black lines) denote the pairwise relatedness between isolates at the selected pairwise allele sharing (*P*_AS_) ≥0.70 threshold (i.e., identical at ≥7 of the 10 microsatellite loci). This threshold was selected to visualise the genetic similarity between isolates that probably resulted from recent transmission and/or recombination events. Note in (b), in Vea/Gowrie there is one isolate pair within the same household denoted with a black circle

To visualize these *P*_AS_ relationships, we constructed spatial networks of the *P*_AS_ scores between infection haplotypes to investigate if the “highly related” isolates (*P*_AS_ ≥ 0.7) clustered at different spatial scales (i.e., at the level of catchment areas and/or households pre‐ or post‐IRS) and/or spatiotemporally (i.e., pre‐ vs. post‐IRS). We observed no apparent geospatial clustering and very few “highly related” haplotypes within pre‐ and post‐IRS (i.e., T1 and T2, Figure 6a,b), or between pre‐ vs. post‐IRS (i.e., T1 vs. T2, Figure 6c). Even when we relaxed our threshold to examine “related” (*P*_AS_ ≥ 0.5) haplotypes, there was no discernible spatial clustering.

#### 3.7.2 Genetic differentiation and fixation

**FIGURE 7:**
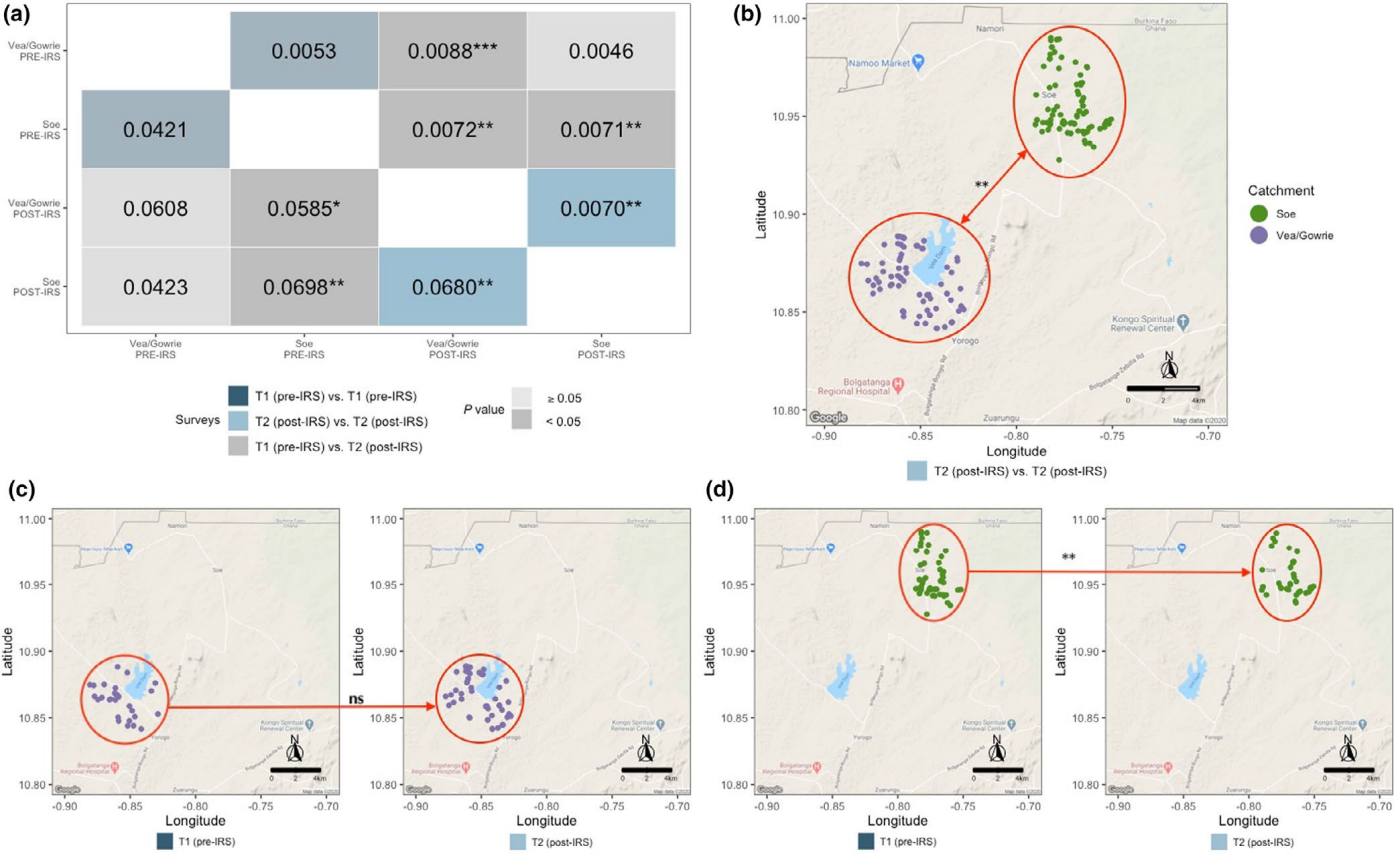
Spatiotemporal genetic differentiation between the *P. falciparum* populations in in Bongo as assessed using the “dominant infections” with complete haplotypes pre‐IRS (T1, October 2012) and post‐IRS (T2, October 2015). (a) Matrix of pairwise *G_ST_* (upper diagonal) and Jost's *D* (lower diagonal) indices between the catchment areas (i.e., Vea/Gowrie and Soe). (b–d) Visualizations of the Jost's *D* pairwise comparisons. Each node represents an isolate and its geographic location in Bongo (i.e., catchment area): purple corresponds to isolates in Vea/Gowrie and green corresponds to isolates in Soe. (b) Vea/Gowrie versus Soe post‐IRS (T2) (Jost's *D* = 0.0680, *p* < .01), (c) Vea/Gowrie pre‐IRS (T1, right panel) to Vea/Gowrie post‐IRS (T2, left panel) (Jost's *D* = 0.0608, *p* ≥ .05, ns), (d) Soe pre‐IRS (T1, right panel) to Soe post‐IRS (T2, left panel) (Jost's *D* = 0.0698, *p* < .01). Note: In (a), grey indicates comparisons between pre‐ versus post‐IRS (T1 vs. T2); dark blue for Vea/Gowrie versus Soe pre‐IRS (T1); light blue for Vea/Gowrie versus Soe post‐IRS (T2). The colour intensity corresponds to the *p*‐value for each pairwise comparison where paler colouring indicates a nonsignificant *p*‐value (*p* ≥ .05) and intense colouring indicates significant *p*‐values (*p* < .05). *p‐value < .05; **p‐value < .01; ***p‐value < .001

To further investigate spatial patterns of genetic differentiation, we calculated the pairwise *G*_ST_ and Jost's pairwise index of differentiation (*D*) over time (i.e., pre‐ to post‐IRS) and over the different spatial scales (i.e., Bongo and the catchment areas) (Figure 7a). Both *G*_ST_ and pairwise Jost's *D* were calculated to measure the extent of allelic fixation and allelic differentiation, respectively (Figure 7a), where Jost's *D* values can be interpreted as the mean proportion of “private alleles” between populations. Allele fixation differences were also found when we compared the allele distributions for each locus independently (Figure 3).

When the pre‐ and post‐IRS surveys were compared, the parasite population in Bongo was genetically differentiated (Jost's *D*: *p* = .034), with significant differences in allelic fixation (*G_ST_*: *p* = .021) (Table S13). In fact, 3.3% [95% CI: 2.1%–4.7%] of alleles across all 10 loci were considered private between the pre‐ and post‐IRS surveys (Table S13). To determine if this temporal genetic differentiation was due to geospatial factors, we next stratified this analysis by catchment area. Using this approach there was clear evidence of spatial genetic differentiation between Vea/Gowrie and Soe, however this was only significant post‐IRS (Jost's *D*: *p* < .01, Figure 7a, lower diagonal; Figure 7b). When we investigated this further, there was no evidence of genetic differentiation in Vea/Gowrie pre‐ to post‐IRS (Jost's *D*: *p* ≥ .05, Figure 7a, lower diagonal; Figure 7c). In contrast, Soe, which shares an immediate border with the Nahouri Province in Burkina Faso to the north, was found to be genetically differentiated pre‐ to post‐IRS (Jost's *D*: *p* < .01, Figure 7a, lower diagonal; Figure 7d). This result indicates that the parasite population in Soe post‐IRS was genetically different from the population that was surveyed pre‐IRS. There was no clear trend in *G_ST_* at the catchment level (Figure 7a, upper diagonal) therefore we could not attribute the significant *G*_ST_ to a specific area and this may reflect the overall differentiation (Jost's *D* values).

#### 3.7.3 Bayesian cluster analysis

To further explore the observed spatiotemporal population structure, a Bayesian cluster analysis was performed. This infers the number of ancestral genetic clusters and assigns each infection haplotype to these clusters. The optimal number of genetic clusters in Bongo and after stratifying by catchment area were identified as *K* = 6 and 7, respectively (pre‐ to post‐IRS, Figure S4). The higher *K* clusters observed from the pre‐ to post‐IRS survey suggests that there were more distinct ancestral haplotypes over time (see Section 2). Nonetheless, all haplotypes segregated into evenly distributed genetic clusters (Figure S5) and there was no evidence of *P. falciparum* subpopulation genetic clustering at any of the temporal or spatial scales investigated (i.e., populations were considered well‐mixed).

## 4 DISCUSSION

Genomic surveillance of putatively neutral variation in microsatellites has proven informative to show that the short‐term IRS intervention against a background of widespread LLIN usage in Bongo, did not bottleneck *Plasmodium falciparum* population diversity and structure. Despite a more than 90% reduction in local transmission and a 35.7% reduction in parasite prevalence pre‐ to post‐IRS, we observed that both genetic diversity and the effective population size of the parasite population increased slightly, rather than reduced. These data highlight the resilience of the parasite population in the human host to vector control. They have relevance for IRS in the many regions of high seasonal transmission typical of sub‐Saharan Africa and a focus of the WHO’s HBHI strategy (World Health Organization & Roll Back Malaria Partnership to End Malaria, 2019).

The obvious resilience of the parasite population in humans to transmission‐reducing interventions like IRS is best explained by the persistence of such infections in the human host. Such infections are generally multiclonal and can last for hundreds of days by the molecular mechanism of clonal antigenic variation (reviewed in Kyes et al., 2007; Miller et al., 2002; Scherf et al., 2008). Our results suggest that the remaining diversity at the end of each dry season is sufficient to maintain the diversity of the parasite population post‐IRS. Furthermore, our data point to gene flow from neighbouring uncontrolled areas as a potential source of increasing diversity. The observed increase in diversity may also be due to reduced outcrossing rates, leading to fewer “related” parasites (i.e., siblings or recent recombinants), and thus higher apparent genetic diversity in the parasite population. In fact, when we examined those isolates collected after the IRS intervention, we observed that they were significantly less likely to be “related” (i.e., limited to no sharing of alleles) compared to those isolates collected pre‐IRS.

Of note, regardless of the intervention, all the multilocus haplotypes constructed for the dominant infections were unique (except for one pair of post‐IRS isolates from the same compound (i.e., household) in Vea/Gowrie, Figure 6b) and virtually all isolate pairs (97.3%) shared less than 50% of their alleles. Moreover, a median of 20% of alleles were shared between haplotypes (i.e., highly unrelated), which was the same as previously observed at the end of the dry season in Bongo (Ruybal‐Pesántez et al., 2017). This confirms our linkage equilibrium findings that IRS has not significantly perturbed outcrossing between unrelated or genetically diverse parasite clones. Moreover, the spatiotemporal *P*_AS_ networks and Bayesian clustering analysis confirmed a lack of clustering among parasites, further supporting free gene flow and a lack of inbred clonal or highly related haplotypes persisting in Bongo.

Typical of high‐transmission areas, there was no evidence of significant multilocus LD both within and between the pre‐ and post‐IRS time points investigated. Although transmission was interrupted, multi‐clonal infections (i.e., MOI > 1) were still prevalent in the population at the end of the wet season post‐IRS. Therefore, sexual recombination between diverse parasites (i.e., outcrossing) still occurred following the IRS intervention, resulting in linkage equilibrium among the 10 microsatellite loci investigated. An alternative explanation for the lack of multilocus LD is that even if outcrossing was reduced following the IRS, continuous gene flow via migration of *P. falciparum* clones from the surrounding uncontrolled areas may have increased genetic diversity but not LD (see discussion below). This contrasts with the surveillance application of microsatellites to detect changes in LD when going from moderate‐ to low‐transmission as observed in low‐transmission areas, including those with perturbations such as LLINs and ACTs ( Carter et al., 2015; Chenet et al., 2012; Kattenberg et al., 2020; Roh et al., 2019). Some pursuing multiple single nucleotide polymorphisms (SNPs) or microhaplotypes for surveillance may speculate that additional loci could increase resolution to detect changes in LD. This is certainly true in low‐transmission. However, we believe our microsatellite result is robust, even with 10 loci in this high‐transmission African setting due to high allelic diversity and extensive haplotype variation.

The high prevalence of multiclonal infections present in high‐transmission areas of sub‐Saharan Africa, like Bongo, limit the opportunity to sample the entire parasite population. Given we did not find a significant reduction, but a slight increase in diversity, it would be reasonable to assume that we have obtained an accurate picture even though restricting sampling to *P. falciparum* isolates with MOI ≤ 2, while excluding those with higher MOI. To date, there is no way around this restricted use of data in high‐transmission settings. Microsatellites do, however, provide a greater capacity to detect a higher number of alleles in the population with relatively few markers so can more accurately capture MOI up to three. Thus, for studies of genetic diversity and population structure in high‐transmission settings, they can be preferable to using multiple SNPs that are only biallelic (Ellegren, 2004; Selkoe & Toonen, 2006).

Consistent with our previous study undertaken in Bongo at end of the dry season in 2012 (Ruybal‐Pesántez et al., 2017), we found no significant geographic differentiation between the catchment areas (i.e., Vea/Gowrie and Soe) at the end of the wet season prior to the introduction of IRS. However, following the IRS, significant *P. falciparum* population structure was detected in Bongo, with the parasite reservoir pre‐IRS being genetically differentiated from the parasite population post‐IRS. Upon further examination this significant population structure was due to both (a) post‐IRS geospatial differences between Vea/Gowrie and Soe, and (b) temporal pre/post‐IRS differences in Soe. The IRS programme may have restricted vector movement between the catchment areas (~15–20 km apart) since Vea/Gowrie and Soe were genetically differentiated from Vea/Gowrie post‐IRS, with approximately 6.8% of alleles being private (Figure 7a). However, given our findings of linkage equilibrium and lack of clustering, another explanation for these results may be due to the proximity of Soe to Burkina Faso, which borders Bongo District to the north (Figure 1a,c). Given that Burkina Faso has a high incidence of malaria, high parasite genetic diversity, and no significant population structure (De Allegri et al., 2013; PMI, 2017; Sondo et al., 2019), human occupational movement and/or vector migration may contribute to continuous gene flow and/or mixing of genetically diverse parasites between these two areas. Since no IRS was undertaken in the Nahouri Province proximal to Soe, the rate of importation of diverse genomes from Burkina Faso is expected to have remained the same over time. The significant reductions in *P. falciparum* prevalence in Soe following the IRS intervention means that these imported infections from Burkina Faso are more likely to be represented in our sampled population. Such observations have been reported within regions of sub‐Saharan Africa (Bei et al., 2018; Duffy et al., 2017; Lynch & Roper, 2011; Mobegi et al., 2012; Roh et al., 2019; Sharp et al., 2007). Moreover, the implementation of control interventions in Burkina Faso has been limited to only 10%–40% population‐wide distribution of LLINs between 2010–2014 (Samadoulougou et al., 2017). In the province that share an immediate border with Bongo District, no IRS programmes have been undertaken (PMI, 2017), therefore, vector and parasite populations in this region were not subjected to the same selection pressures as those in Bongo.

These results indicate that, in high‐transmission settings, short‐term IRS interventions alone will not be sufficient to reduce parasite diversity. IRS will probably need to be sustained or combined with chemotherapeutic interventions (e.g., mass drug administration) to achieve low‐transmission and bottleneck the parasite population in the human population in high‐transmission areas like Bongo. Further investigation into these trends would provide insight into the threshold needed for perturbations to have a substantial effect on neutral diversity in these high‐transmission settings where the *P. falciparum* reservoir remains highly diverse (He & Pascual, 2020). Our data show that future vector control interventions conducted in high‐transmission settings in sub‐Saharan Africa, will benefit by incorporating molecular surveillance to assess progress towards achieving the WHO Global Technical Strategy for Malaria 2016–2030 targets (World Health Organization, 2015a).

## AUTHOR CONTRIBUTIONS

M.P., K.A.K. and K.P.D. conceived and designed the study. A.R.O., S.K.D., M.A.A., V.A. and K.E.T. contributed to the study design and co‐ordinated the field studies. D.C.A., S.R.‐P., S.L.D. and K.E.T. processed the samples. D.C.A., S.R.P. and S.L.D. conducted the microsatellite genotyping. D.C.A., S.R.‐P. and K.E.T. analysed the data. D.C.A. wrote the original draft of the manuscript. S.R.‐P., K.P.D. and K.E.T. critically revised the manuscript. A.R.O., M.P., K.A.K. and K.P.D. acquired funding.

## Supporting information

Supplementary Material

## Data Availability

The data that support the findings of this study have been made openly available in Dryad at https://doi.org/10.5061/dryad.kh189324z
