# Supplementary material for: The impact of indoor residual spraying on Plasmodium falciparum microsatellite variation in an area of high seasonal malaria transmission in Ghana, West Africa
Source: Mol Ecol. 2021 Jul 16;30(16):3974–92. doi: 10.1111/mec.16029 (PMC8456823; doi:10.1111/mec.16029)
Supplement: Supplementary file 1 — Supplementary Material [file MEC-30-3974-s001.pdf]

MOLECULAR ECOLOGY

Supplemental Information for:

The impact of indoor residual spraying on *Plasmodium falciparum* microsatellite variation in an area of high seasonal malaria transmission in Ghana, West Africa

Dionne C. Argyropoulos, Shazia Ruybal-Pesántez, Samantha L. Deed, Abraham R. Oduro, Samuel K. Dadzie, Maxwell A. Appawu, Victor Asoala, Mercedes Pascual, Kwadwo A. Koram, Karen P. Day, Kathryn E. Tiedje

Table of Contents:

Supplemental Tables	
Table S1	Page 2
Table S2	Page 3
Table S3	Page 4
Table S4	Page 5
Table S5	Page 6
Table S6	Page 7
Table S7	Page 8
Table S8	Page 9
Table S9	Page 11
Table S10	Page 12
Table S11	Page 13
Table S12	Page 14
Table S13	Page 15
**Supplemental Figures**	
Figure S1	Page 16
Figure S2	Page 17
Figure S3	Page 18
Figure S4	Page 19
Figure S5	Page 20
**Supplemental Methods**	Page 21
**References**	Page 23

SUPPLEMENTAL TABLES

Table S1. Participant reported intervention use pre-IRS (T1, October 2012) and post-IRS (T2, October 2015) for the whole population surveyed and for those participants with microscopic *P. falciparum* infections.

Characteristic	Pre-IRS (T1, Oct 2012)	Post-IRS (T2, Oct 2015)	*P-value*
**Total Population ***	1923	2022	
Reported LLIN usage †			0.126
Yes	1713 (89.1)	1831 (90.6)	
No	210 (10.9)	191 (9.4)	
Reported antimalarial usage ‡			<0.001
Yes	796 (41.4)	298 (14.7)	
No	1127 (58.6)	1724 (85.3)	
**Microscopic *P. falciparum* infections ***	808	545	
Reported LLIN usage †			0.857
Yes	712 (88.1)	482 (91.2)	
No	96 (11.9)	63 (8.8)	
Reported antimalarial usage ‡			<0.001
Yes	322 (39.9)	74 (13.6)	
No	486 (60.1)	471 (86.4)	

LLIN = long-lasting insecticidal nets
* Data reflect No. (% (*n*/*N*)) of participants.
† Participant self-reported LLIN usage the previous night
‡ Participant self-reported antimalarial treatment in the two-weeks prior to being surveyed.

Table S2. Microsatellite genotyping success for pre-IRS (T1, N=200) and post-IRS (T2, N=200).

		Genotype Success (%)	
Marker	Chr	Pre-IRS (T1)	Post-IRS (T2)
POLYA	4	88.9	97.0
TA81	5	94.0	95.0
TA42	5	69.9	26.5
TA1	6	69.0	86.0
TA109	6	98.1	98.0
TA87	6	95.8	98.5
TA40	10	78.2	96.0
2490	10	85.6	78.5
ARA2	11	88.9	80.0
PFG377	12	93.1	89.5
PFPK2	12	92.6	96.0
TA60	13	90.3	90.5
Median		89.6	92.8

Table S3. Demographics and parasitological characteristics of the study population in Bongo collected for the pre-IRS (T1, October 2012) and post-IRS (T2, October 2015) surveys.

Characteristic	Pre-IRS (T1, Oct 2012)	Post-IRS (T2, Oct 2015)	*P-value*
**Total Population**	1923	2022	
Age Groups *			0.221
1 – 5 years	356 (18.5)	405 (20.0)	
6 – 10 years	395 (20.5)	409 (20.2)	
11 – 20 years	413 (21.5)	467 (23.1)	
21 – 39 years	326 (17.0)	297 (14.7)	
≥ 40 years	433 (22.5)	444 (22.0)	
Sex *			0.781
Female	1031 (53.6)	1093 (54.1)	
Male	892 (46.4)	929 (45.9)	
Catchment Area *			0.295
Vea/Gowrie	919 (47.8)	1000 (49.5)	
Soe	1004 (52.2)	1022 (50.5)	
P. falciparum* prevalence *			
All	808 (42.0)	545 (27.0)	<0.001
Age Groups			<0.001
1 – 5 years	173 (48.6)	63 (15.6)	
6 – 10 years	243 (61.5)	167 (40.8)	
11 – 20 years	202 (48.9)	169 (36.2)	
21 – 39 years	84 (25.8)	52 (17.5)	
≥ 40 years	106 (24.5)	94 (21.2)	
Sex			0.376
Female	379 (36.8)	269 (24.6)	
Male	429 (48.1)	276 (29.7)	
Catchment Area			0.165
Vea/Gowrie	356 (35.5)	261 (26.1)	
Soe	452 (45.0)	284 (27.8)	
***Plasmodium* spp. median density †**			
All	520 [160 – 1640]	320 [120 – 1800]	<0.001
Age Groups			<0.001
1 – 5 years	1640 [400 – 9860]	1840 [260 – 19940]	
6 – 10 years	720 [240 – 1840]	520 [200 – 2720]	
11 – 20 years	320 [160 – 760]	280 [120 – 1000]	
21 – 39 years	200 [120 – 720]	200 [80 – 1730]	
≥ 40 years	200 [120 – 670]	120 [40 – 320]	
Sex			0.006
Female	520 [160 – 1640]	320 [80 – 1960]	
Male	480 [160 – 1610]	320 [120 – 1300]	
Catchment			<0.001
Vea/Gowrie	360 [160 – 1190]	240 [80 – 1280]	
Soe	680 [200 – 2180]	400 [120 – 2210]	
***P. falciparum* median MOI ‡**			
All	3 [1-4]	1 [1-2]	<0.001
Age Groups			<0.001
1 – 5 years	3 [2-5]	2 [1-2]	
6 – 10 years	3 [2-5]	2 [1-3]	
11 – 20 years	2 [1-4]	2 [1-2]	
21 – 39 years	2 [1-2.25]	1 [1-2]	
≥ 40 years	1 [1-2]	1 [1-1]	
Sex			<0.001
Female	2 [1-4]	1 [1-2]	
Male	3 [1-4]	1.5 [1-2]	
Catchment			<0.001
Vea/Gowrie	2 [1-4]	1 [1-2]	
Soe	3 [2-4]	2 [1-3]	

IQR = inter quartile range; MOI= multiplicity of infection

* Data reflect No. (% (n/N) participants.

** Data reflect No. (% (*n*/*N*)) of participants that were positive for *P. falciparum* (and mixed *P. falciparum*/*P. malariae*) by microscopy.

† Median parasite density for the microscopically positive *P. falciparum* (including mixed P. falciparum/P. malariae infections) (value/ μL [IQR]) isolates.

‡ Data reflect the median estimated MOI based on *var* genotyping (see Supplemental Methods).

Table S4. The microscopic *P. falciparum* isolates selected for the microsatellite analyses in the pre-IRS (T1, October 2012) and post-IRS (T2, October 2015) surveys. Statistical analyses were completed to determine if there were any significant differences, with respect to any of the study variables, between those isolates selected based on MOI and those excluded.

**Characteristic**	**Pre-IRS (T1, Oct 2012)**		**Post-IRS (T2, Oct 2015)**	
	N *	n **	N *	n **
**All *P. falciparum* infections**	808	192 (23.7)	545	200 (36.7)
Age groups				
1 – 5 years	173 (21.4)	36 (20.8)	63 (11.6)	17 (27.0)
6 – 10 years	243 (30.1)	37 (15.2)	167 (30.6)	68 (40.7)
11 – 20 years	202 (25.0)	52 (25.7)	169 (31.0)	58 (34.3)
21 – 39 years	84 (10.4)	27 (32.1)	52 (9.5)	20 (38.5)
≥ 40 years	106 (13.1)	40 (37.7)	94 (17.3)	37 (39.4)
P-value		<0.001		0.707
Sex				
Female	379 (46.9)	101 (26.6)	269 (49.4)	98 (36.4)
Male	429 (53.1)	91 (21.2)	276 (50.6)	102 (37.0)
P-value		0.155		0.931
Catchment Area				
Vea/Gowrie	356 (44.1)	87 (24.4)	261 (47.9)	103 (39.5)
Soe	452 (55.9)	105 (23.2)	284 (52.1)	97 (34.2)
P-value		0.753		0.382
***Plasmodium* spp. median density †**				
All	520 [160 – 1640]	240 [120 – 2020]	320 [120 – 1800]	320 [120 – 1190]
P-value		0.7841		0.008
Age Groups				
1-5 Years	1640 [400 – 9860]	6480 [230 – 46620]	1840 [260 – 19940]	1640 [320 – 17280]
6-10 Years	720 [240 – 1840]	360 [120 – 1480]	520 [200 – 2720]	360 [160 – 1430]
11-20 Years	320 [160 – 760]	160 [80 – 610]	280 [120 – 1000]	340 [160 – 1060]
21-39 Years	200 [120 – 720]	160 [80 – 740]	200 [80 – 1730]	320 [120 – 2200]
40+ Years	200 [120 – 670]	220 [110 – 1150]	120 [40 – 320]	120 [80 – 480]
Sex				
Female	520 [160 – 1640]	240 [120 – 1560]	320 [80 – 1960]	320 [120 – 1520]
Male	480 [160 – 1610]	240 [120 – 2940]	320 [120 – 1300]	360 [160 – 1080]
Catchment Areas				
Vea/Gowrie	360 [160 – 1190]	200 [120 – 1140]	240 [80 – 1280]	360 [160 – 1120]
Soe	680 [200 – 2180]	400 [120 – 2400]	400 [120 – 2210]	320 [120 – 1280]

* Data reflect No. (% N/Total number microscopically positive *P. falciparum* isolates in T1 or T2) of microscopically positive *P. falciparum* isolates.

** Data reflect No. (% (*n*/*N*)) of microscopically positive *P. falciparum* isolates selected for T1 and T2.

P-values reflect chi-squared test comparison of the *P. falciparum* positive population N=808 (T1) and N=545 (T2) to the N=192 (T1) and N=200 (T2) microsatellite-selected population.

† Median parasite density for the microscopically positive *P. falciparum* (including mixed P. falciparum/P. malariae infections) (value/ μL [IQR]) isolates. *P-values* reflect Kruskal-Wallis test comparison of the *P. falciparum* positive population N=808 (T1) and N=545 (T2) to the N=192 (T1) and N=200 (T2) microsatellite-selected population.

Table S5. Patterns of genetic diversity for the *P. falciparum* populations from Bongo for the "all infections" dataset in the pre-IRS (T1, October 2012) and post-IRS (T2, October 2015) surveys.

	n		*h*		*A*			R_s			H_e		
Population	T1	T2	T1	T2	T1	T2	*P-value**	T1	T2	*P-value**	T1	T2	*P-value***
Vea/Gowrie	87	104	87	103	10.5	11.4	0.89	8.9	10.6	0.96	0.78	0.81	0.01
Soe	105	96	105	96	10.8	11.0	0.98	8.6	10.3	0.98	0.78	0.80	0.12
Total	192	200	192	199	12.0	12.9	0.90	10.8	12.4	0.94	0.78	0.81	0.01

n = number of isolates; *h* = number of haplotypes; *A* = mean number of alleles per locus;
R_s = allelic richness estimate; H_e = expected heterozygosity.
P-value = T1 and T2 comparison. **P*-values calculated by chi-square test. ***P*-value calculated by "Hs.test" function.

Table S6. Allelic size range, number of alleles, private alleles and allele frequency for the "all infections" dataset for the 10 microsatellite loci in the pre-IRS (T1, October 2012) and post-IRS (T2, October 2015) surveys.

		N		Allele Range		Alleles per locus		Private Alleles		Allele Frequency
Marker	Chr	T1	T2	T1	T2	T1	T2	T1	T2	*P-value*
POLYA	4	173	194	133 – 184	112 – 190	18	22	1	5	0.310
TA81	5	185	190	104 – 134	104 – 137	11	12	0	1	0.680
TA109	6	190	196	148 – 199	148 – 217	17	17	2	2	0.870
TA87	6	188	197	74 – 116	71 – 122	11	14	1	4	0.200
TA40	10	152	192	203 – 290	209 – 266	20	18	5	3	0.900
2490	10	167	157	74 – 89	74 – 89	5	5	0	0	0.660
ARA2	11	172	160	60 – 84	60 – 87	9	10	0	1	0.014*
PFG377	12	182	179	89 – 104	89 – 104	6	6	0	0	<0.001***
PFPK2	12	181	192	152 – 188	152 – 197	13	14	0	1	0.860
TA60	13	176	181	68 – 95	65 – 98	10	11	1	2	<0.001***

Genetic characteristics of 392 infections ("all infections" dataset) using 10 microsatellite loci from T1 (*N* = 192) and T2 (*N* = 200).
N = number of isolates.
Private Allele comparisons are between T1 to T2. Allele Frequency comparison by Mann-Whitney U Test. *** $P < 0.001$, * $P < 0.05$.

Table S7. Allelic size range, number of alleles, private alleles and allele frequency for the "dominant infections" dataset for the 10 microsatellite loci in the pre-IRS (T1, October 2012) and post-IRS (T2, October 2015) surveys.

		N		Allele Range		Alleles per locus		Private Alleles		Allele Frequency
Marker	Chr	T1	T2	T1	T2	T1	T2	T1	T2	*P-value*
POLYA	4	115	151	136 – 184	112 – 190	17	21	1	5	0.190
TA81	5	123	149	104 – 154	107 – 137	11	11	1	1	0.340
TA109	6	126	153	148 – 199	154 – 217	17	15	3	2	0.850
TA87	6	124	153	74 – 116	71 – 122	11	14	1	4	0.050*
TA40	10	109	150	203 – 290	215 – 266	19	17	5	3	0.800
2490	10	110	127	74 – 89	74 – 89	5	5	0	0	0.200
ARA2	11	112	123	60 – 81	60 – 87	8	10	0	2	0.040*
PFG377	12	119	143	89 – 104	89 – 104	6	6	0	0	<0.001***
PFPK2	12	121	151	152 – 188	152 – 197	13	14	0	1	0.740
TA60	13	117	140	71 – 95	65 – 98	8	11	0	3	0.030*

Genetic characteristics of 284 infections ("dominant infections" dataset) using 10 microsatellite loci from T1 (N = 128) and T2 (N = 156).
N = number of isolates.
Private Allele comparisons are between T1 to T2. Allele Frequency comparison by Mann-Whitney U Test. ***$P < 0.001$, **$P < 0.01$, *$P < 0.05$.

Table S8. Allele frequencies at 10 microsatellite loci for "all infections" and "dominant infections" datasets in the pre-IRS (T1, October 2012) and post-IRS (T2, October 2015) surveys.

		All Infections		Dominant Infections	
Marker	bp	Pre-IRS (T1)	Post-IRS (T2)	Pre-IRS (T1)	Post-IRS (T2)
POLYA		*N*=173	*N*=194	*N*=115	*N*=151
	112	-	0.005	-	0.007
	115	-	0.010	-	0.013
	130	-	0.010	-	0.013
	133	0.006	-	-	-
	136	0.017	0.036	0.017	0.033
	139	0.023	0.021	0.017	0.013
	142	0.029	0.026	0.026	0.026
	145	0.035	0.046	0.035	0.046
	148	0.069	0.067	0.070	0.066
	151	0.168	0.196	0.148	0.185
	154	0.179	0.113	0.174	0.113
	157	0.075	0.103	0.104	0.113
	160	0.087	0.088	0.061	0.099
	163	0.110	0.093	0.122	0.079
	166	0.052	0.031	0.052	0.033
	169	0.029	0.052	0.035	0.040
	172	0.040	0.031	0.026	0.046
	175	0.023	0.031	0.035	0.026
	178	0.029	0.005	0.035	-
	181	0.012	0.010	0.017	0.013
	184	0.017	0.005	0.026	0.007
	187	-	0.015	-	0.020
	190	-	0.005	-	0.007
TA81		*N*=185	*N*=190	*N*=123	*N*=149
	104	0.005	0.0053	0.008	-
	107	0.011	0.005	0.008	0.007
	110	0.022	0.005	0.024	0.007
	113	0.032	0.047	0.033	0.047
	116	0.168	0.226	0.197	0.242
	119	0.222	0.211	0.195	0.228
	122	0.324	0.247	0.341	0.248
	125	0.059	0.074	0.065	0.081
	128	0.103	0.137	0.081	0.107
	131	0.038	0.032	0.041	0.020
	134	0.016	0.005	0.024	0.007
	137	-	0.005	-	0.007
TA109		*N*=190	*N*=196	*N*=126	*N*=153
	148	0.021	0.005	0.008	-
	151	-	-	-	-
	154	0.011	0.005	0.016	0.007
	157	0.005	-	0.008	-
	160	0.216	0.235	0.198	0.216
	163	0.127	0.143	0.143	0.150
	166	0.005	0.026	0.008	0.033
	169	0.011	0.010	0.016	0.007
	172	0.184	0.153	0.206	0.163
	175	0.247	0.281	0.230	0.275
	178	0.032	0.015	0.032	0.020
	181	0.005	0.020	0.008	0.026
	184	0.016	-	0.024	-
	187	0.011	0.026	0.016	0.026
	190	0.011	0.005	0.016	-
	193	0.005	0.055	0.008	0.007
	196	0.074	0.051	0.048	0.046
	199	0.021	0.010	0.016	0.013
	208	-	-	-	-
	214	-	0.005	-	0.007
	217	-	0.005	-	0.007
TA87		*N*=188	*N*=197	*N*=124	*N*=153
	71	-	0.005	-	0.007
	74	0.011	-	0.016	-
	80	-	-	-	-
	83	-	-	-	-
	86	-	0.011	-	0.007
	89	0.032	0.051	0.040	0.052
	92	0.043	0.020	0.048	0.026
	95	0.202	0.127	0.226	0.118
	98	0.133	0.1422	0.161	0.131
	101	0.202	0.228	0.161	0.229
	104	0.165	0.173	0.121	0.190
	107	0.138	0.142	0.153	0.144
	110	0.053	0.051	0.048	0.039
	113	0.011	0.025	0.016	0.026
	116	0.011	0.015	0.008	0.020
	119	-	0.005	-	0.007
	122	-	0.005	-	0.007
	125	-	-	-	-
TA40		*N*=152	*N*=192	*N*=109	*N*=150
	203	0.007	-	0.009	-
	206	-	-	-	-
	209	0.007	0.016	-	-
	212	0.007	-	0.009	-

Continued next page.

	215	0.026	0.016	0.009	0.020
	218	-	0.005	-	0.007
	221	0.059	0.104	0.073	0.087
	224	0.053	0.031	0.064	0.033
	227	0.079	0.099	0.083	0.107
	230	0.007	0.016	0.009	0.007
	233	0.013	0.016	0.018	0.013
	236	0.020	0.010	0.009	0.013
	239	0.039	0.042	0.037	0.053
	242	0.224	0.172	0.211	0.173
	245	0.263	0.177	0.257	0.193
	248	0.046	0.172	0.046	0.167
	251	0.026	0.031	0.018	0.033
	254	0.072	0.047	0.083	0.040
	257	-	0.021	-	0.027
	260	0.026	0.021	0.028	0.020
	263	0.013	-	0.018	-
	266	-	0.005	-	0.007
	272	-	-	-	-
	281	0.007	-	0.009	-
	287	-	-	-	-
	290	0.007	-	0.009	-
2490		*N*=167	*N*=157	*N*=110	*N*=127
	74	0.024	0.045	0.018	0.039
	80	0.240	0.261	0.291	0.244
	83	0.689	0.055	0.636	0.559
	86	0.018	0.121	0.027	0.126
	89	0.030	0.025	0.027	0.031
ARA2		*N*=172	*N*=160	*N*=112	*N*=123
	60	0.105	0.081	0.134	0.089
	63	0.076	0.069	0.080	0.089
	66	0.302	0.250	0.304	0.252
	69	0.186	0.138	0.188	0.163
	72	0.163	0.163	0.161	0.138
	75	0.105	0.138	0.080	0.114
	78	0.029	0.088	0.018	0.089
	81	0.029	0.031	0.038	0.033
	84	0.006	0.025	-	0.024
	87	-	0.019	-	0.008

PFG377		*N*=182	*N*=179	*N*=119	*N*=143
	89	0.027	0.006	0.034	0.007
	92	0.099	0.034	0.101	0.028
	95	0.264	0.218	0.277	0.189
	98	0.549	0.553	0.513	0.587
	101	0.038	0.078	0.042	0.077
	104	0.022	0.112	0.034	0.112
PFPK2		*N*=181	*N*=192	*N*=121	*N*=151
	152	0.006	0.010	0.008	0.013
	155	0.017	0.016	0.008	0.013
	158	0.105	0.120	0.107	0.132
	161	0.166	0.182	0.140	0.152
	164	0.309	0.245	0.331	0.238
	167	0.133	0.151	0.116	0.172
	170	0.088	0.104	0.091	0.106
	173	0.050	0.057	0.066	0.060
	176	0.033	0.026	0.041	0.033
	179	0.033	0.042	0.025	0.040
	182	0.022	0.021	0.025	0.020
	185	0.022	0.016	0.025	0.007
	188	0.017	0.005	0.017	0.007
	191	-	-	-	-
	194	-	-	-	-
	197	-	0.005	-	0.007
TA60		*N*=176	*N*=181	*N*=117	*N*=140
	65	-	0.028	-	0.029
	68	0.017	-	-	-
	71	0.244	0.127	0.231	0.136
	74	0.233	0.149	0.222	0.157
	77	0.028	0.055	0.034	0.043
	80	0.239	0.298	0.231	0.279
	83	0.176	0.177	0.222	0.186
	86	0.028	0.110	0.017	0.107
	89	0.017	0.017	0.026	0.021
	92	0.006	0.011	-	0.007
	95	0.011	0.011	0.017	0.014
	98	-	0.017	-	0.021

Table S9. Patterns of genetic diversity for the *P. falciparum* populations per catchment area for the "dominant infections" dataset by locus in the pre-IRS (T1, October 2012) and post-IRS (T2, October 2015) surveys.

Locus	Pre-IRS (T1, October 2012) H_e		Post-IRS (T2, October 2015) H_e	
	Vea/Gowrie	Soe	Vea/Gowrie	Soe
POLYA	0.916	0.902	0.918	0.898
TA81	0.825	0.782	0.797	0.821
TA109	0.813	0.865	0.836	0.788
TA87	0.855	0.835	0.861	0.842
TA40	0.865	0.845	0.849	0.888
2490	0.446	0.542	0.656	0.553
ARA2	0.832	0.807	0.873	0.830
PFG377	0.697	0.611	0.570	0.631
PFPK2	0.819	0.841	0.866	0.849
TA60	0.772	0.801	0.807	0.847
Overall	0.784	0.783	0.803	0.794

H_e = expected heterozygosity.
Genetic characteristics of 284 infections ("dominant infections" dataset) using 10 microsatellite loci from T1 (N = 128) and T2 (N = 156). The "overall" row refers to the total H_e for the catchment area. See Figure 1A for location of Vea/Gowrie and Soe catchment areas.

Table S10. Population bottleneck analysis by locus for the two survey time points using the Stepwise-Mutation Model (SMM) in in the pre-IRS (T1, October 2012) and post-IRS (T2, October 2015) surveys.

		Pre-IRS (T1)			Post-IRS (T2)		
Locus	Model	H_e	SMM		H_e	SMM	
POLYA	SMM	0.907	0.468	E	0.912	0.174	D
TA81	IAM	0.802	0.092	D	0.810	0.105	D
TA109	SMM	0.843	0.005	D	0.826	0.012	D
TA87	IAM	0.855	0.470	E	0.857	0.130	D
TA40	SMM	0.864	0.001	D	0.880	0.109	D
2490	IAM	0.511	0.072	D	0.612	0.256	D
ARA2	IAM	0.818	0.300	E	0.856	0.245	E
PFG377	IAM	0.649	0.165	D	0.602	0.085	D
PFPK2	IAM	0.833	0.064	D	0.857	0.123	D
TA60	IAM	0.796	0.479	E	0.832	0.283	D
Overall			0.984			0.999	

H_e: Heterozygosity; H_e excess (*E*) is the transient increase in the expected heterozygosity compared to that observed for the population, while H_e deficiency (*D*) is a lower expected heterozygosity than observed. *P* value tests an excess of H_e via a Wilcoxon signed-rank test calculated by BOTTLENECK v. 1.2.02.

Table S11. Number of loci with excess or deficiency in the heterozygosity (*H_e*) relative to the heterozygosity at mutation-drift equilibrium (*H_{eq}*) in the *P. falciparum* populations in the Bongo catchment areas in the pre-IRS (T1, October 2012) and post-IRS (T2, October 2015) surveys.

Population	H_e excess	H_e deficiency	*P-value*	Mode shift
Pre-IRS (T1, October 2012)				
Vea/Gowrie	4	6	0.862	Normal
Soe	4	6	0.947	Normal
Post-IRS (T1, October 2015)				
Vea/Gowrie	2	8	0.947	Normal
Soe	4	6	0.947	Normal

P-value test as an excess of H_e calculated as a Wilcoxon sign-rank test; Normal: L-shaped distribution = non-bottlenecked population, Shifted: shifted mode = bottlenecked population.

NOTE: H_e excess is the transient increase in H_{eq} compared to H_e observed for the population, while H_e deficiency is a lower H_{exp} than H_e observed. These data were subject to mutation drift equilibrium via SMM and mode-shift analyses (BOTTLENECK v. 1.2.02).

Table S12. Number of Pairwise Allele Sharing (P_{AS}) comparisons within (i.e., pre- and post-IRS) and between (i.e., pre- vs. post-IRS) the time point surveys investigated for the "dominant infections" with complete haplotypes.

P_{AS} score	Pre-IRS (T1)	Post-IRS (T2)	Pre- vs. Post-IRS (T1 vs. T2)
Number of pairwise comparisons	3,240	3,486	6,804
0.0	295 (9.1)	370 (10.6)	669 (9.8)
0.1	831 (25.6)	979 (28.1)	1835 (27.0)
0.2	992 (30.6)	1106 (31.7)	2141 (31.5)
0.3	726 (22.4)	665 (19.1)	1428 (21.0)
0.4	298 (9.2)	273 (7.8)	559 (8.2)
0.5	68 (2.1)	78 (2.2)	135 (2.0)
0.6	25 (0.8)	12 (0.3)	34 (0.5)
0.7	3 (0.09)	1 (0.02)	3 (0.04)
0.8	2 (0.06)	1 (0.02)	-
0.9	-	-	-
1.0	-	1 (0.02)	-
Median P_{AS} [min – max]	0.2 [0.0 – 0.8]	0.2 [0.0 – 1.0]	0.2 [0.0 – 0.7]

* Data reflect No. (% (*n*/*N*)) of pairwise comparisons within each group, i.e., T1, T2 and T1 vs. T2.

Table S13. Genetic differentiation (G_{ST} and Jost's *D*) of *P. falciparum* populations from Bongo in in the pre-IRS (T1, October 2012) and post-IRS (T2, October 2015) surveys by locus for the "dominant infections" dataset.

		Pre- to Post-IRS (T1 to T2)			
Locus	Chr	G_{ST}	*P*-value	Jost's *D*	*P-value*
POLA	4	0.0014	0.542	0.0291	0.519
TA81	5	0.0031	0.236	0.0258	0.244
TA109	6	0.0002	0.770	0.0016	0.790
TA87	6	0.0050	0.081	0.0595	0.073
TA40	10	0.0054	0.057	0.0739	0.054
2490	10	0.0060	0.131	0.0155	0.130
ARA2	11	0.0017	0.503	0.0172	0.510
PFG377	12	0.0087	0.041*	0.0293	0.036*
PFPK2	12	0.0021	0.376	0.0235	0.355
TA60	13	0.0061	0.076	0.0539	0.067
Mean		0.00397 [0.0026 – 0.0058]	0.021*	0.0329 [0.0209 – 0.0473]	0.034*

[ ]: 95% confidence interval; **P*<0.05

SUPPLEMENTAL FIGURES.

	T1 Pre IRS, October 2012	T2 Post-IRS, October 2015	Total
All participants	*N* = 1,923	*N* = 2,022	*N* = 3,945
Plasmodium falciparum microscopy positive participants	*N* = 808	*N* = 545	*N* = 1,353
Isolates with selected microsatellite genotyping*	*N* = 200	*N* = 200	*N* = 400
Isolates included for microsatellite genotyping analyses ("all infections" dataset) ⱡ	*N* = 192	*N* = 200	*N* = 392
"Dominant infections" dataset (MOI 1 or 2)‡	*N* = 128	*N* = 156	*N* = 284
"Dominant infections" with complete haplotypes∝	*N* = 81	*N* = 84	*N* = 165

Figure S1. Data used for epidemiological and population genetics analysis.

*Selected *N* = 200 isolates with an MOI = 1 or 2 based on *var* genotyping (see Supplemental Methods).
ⱡ Multilocus haplotypes with greater than or equal to three alleles at the 10 microsatellite loci with > 75% genotype success.
‡ MOI based on the number of clones detected by microsatellite genotyping.
∝ Haplotypes were considered "complete" if there was an allele at each of the 10 microsatellite loci.

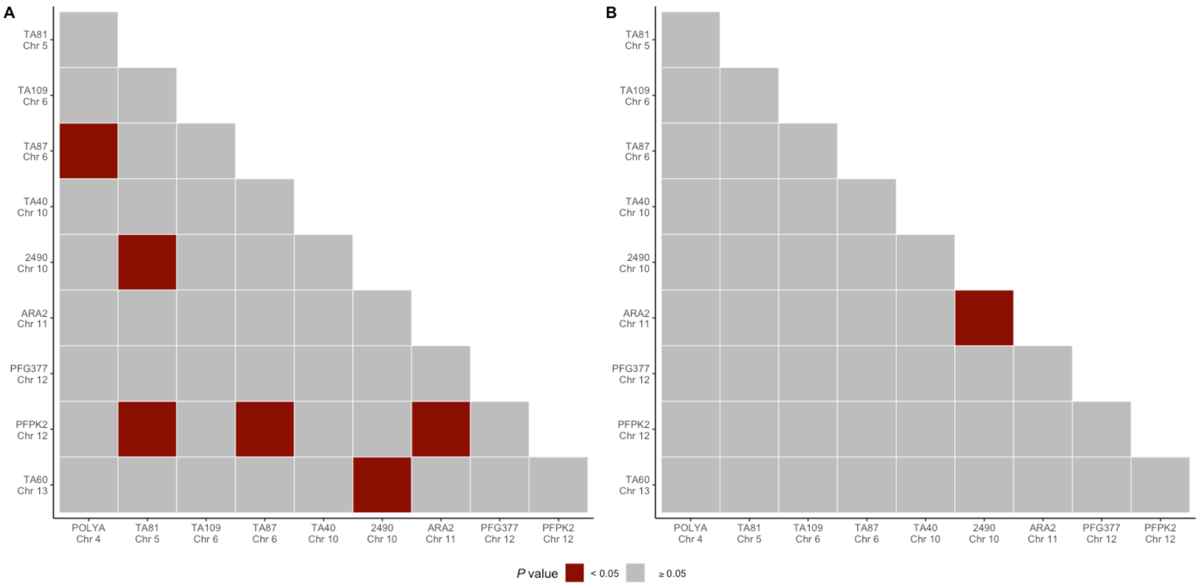

Figure S2. Pairwise linkage disequilibrium ($\bar{r}_d$) for dominant infections with complete haplotypes in **(A)** Pre-IRS (T1, October 2012) (N = 81) and **(B)** Post-IRS (T2, October 2015) (N = 84). The colour key provided corresponds to the *P* value for each pairwise comparison where grey indicates a non-significant *P* value ($P > 0.05$) and red represents significant *P* values ($P < 0.05$).

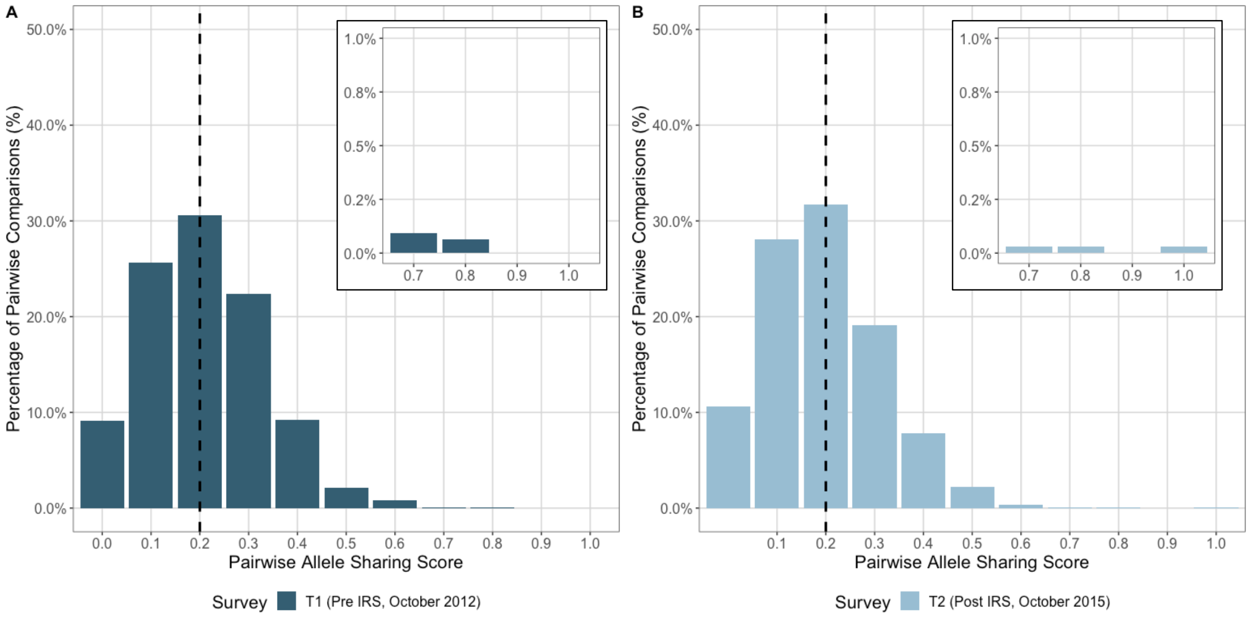

Figure S3. Distribution of the pairwise allele sharing (P_{AS}) scores between the dominant infections with complete haplotypes in **(A)** Pre-IRS (T1, October 2012) and **(B)** Post-IRS (T2, October 2015). The P_{AS} score is represented as a proportion (in %) of the number of alleles shared between two haplotypes divided by the total number of loci considered (i.e., 10) (see Materials and Methods). The median was $P_{AS} = 0.2$ in both pre- and post-IRS survey and is represented by the black dotted line. The P_{AS} scores between 0.7 to 1.0 are shown in the upper right insert. Pre-IRS (T1) there were 81 haplotypes and 3,240 pairwise comparisons, and post-IRS (T2) there were 84 haplotypes and 3,486 pairwise comparisons.

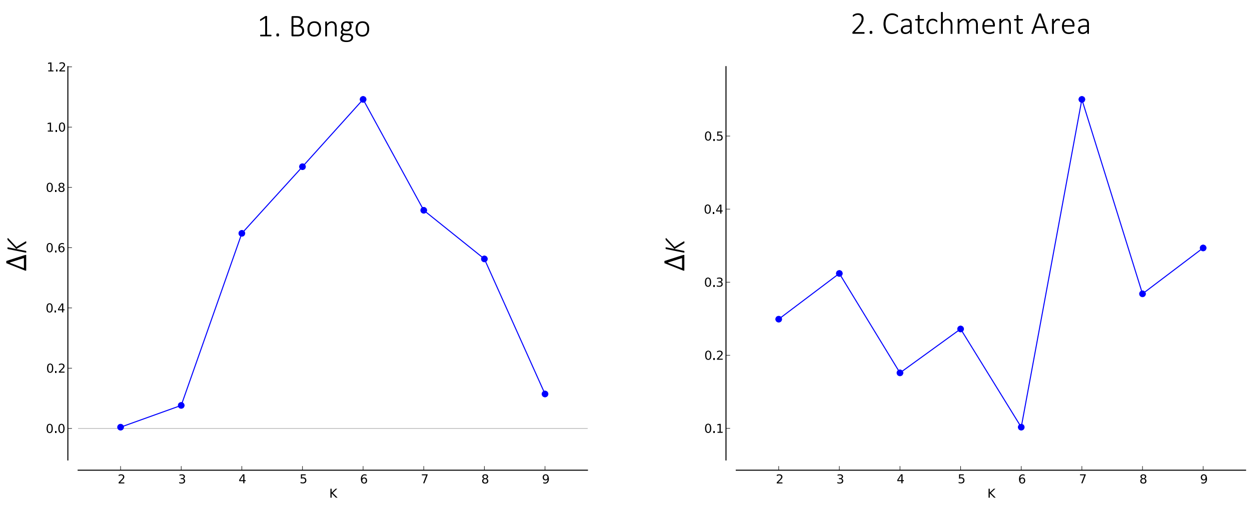

Figure S4. Δ*K* values generated from STRUCTURE Harvester v. 0.6.94 (Earl and vonHoldt, 2012). The highest Δ*K* value from the pre- to post-IRS time points (T1 to T2) and spatial levels (1. Bongo and 2. Catchment Area) were used to plot the optimal *K* clusters in Figure S5.

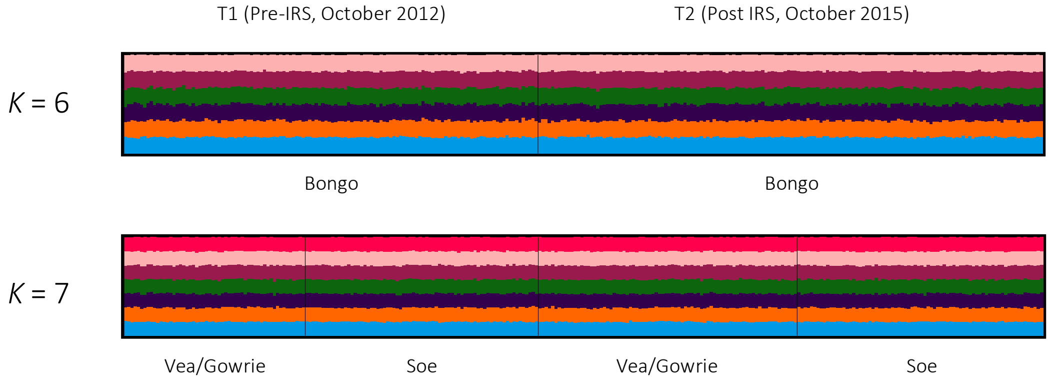

Figure S5. Bayesian cluster analysis of the *P. falciparum* microsatellite haplotypes from Bongo over time. STRUCTURE v. 2.3.4 software (Pritchard et al., 2000) was used to find and cluster populations within the dominant *P. falciparum* infections with complete haplotypes pre-IRS (T1, October 2012) and post-IRS (T2, October 2015). The analyses were run for *K* genetic clusters and 100,000 Markov chain Monte Carlo (MCMC) iterations, after a burn-in period of 100,000 using the admixture model and correlated allele frequencies. *P. falciparum* haplotypes were assigned to a defined number of genetic clusters (*K*) based on genetic distance, where the optimal *K* found by the Evanno *et al.* (2005) (Evanno, Regnaut, & Goudet, 2005) method is represented on the left-hand side. Vertical bars represent individual *P. falciparum* haplotypes, and the colours represent the ancestry co-efficient (Q) for each genetic cluster.

SUPPLEMENTAL METHODS

Entomology. Monthly entomological surveys were undertaken during the wet and dry seasons between February 2013 and September 2015 to monitor the impacts of IRS on the vector population and change in transmission. The main vector in Bongo was *Anopheles gambiae* s.l with limited transmission from *Anopheles funest*us. Pre-IRS, at the peak of the wet-season in August 2013, the daily human biting-rates (HBR; bites/person/night (b/p/n))) were 39.5 b/p/n and 11.4 b/p/n in Vea/Gowrie and Soe, respectively. Post-IRS in August 2015, the daily HBR declined by ~91% and ~99% in Vea/Gowrie (3.5 b/p/n) and Soe (0.1 b/p/n), respectively. The pre-IRS entomological inoculation rates (EIR) (i.e., infective bites/person/month) in August 2013 were 5.9 and 10.7 in Vea/Gowrie and Soe, respectively. After the three-rounds of IRS in August 2015, EIR decreased by ~99% and ~93% in Vea/Gowrie (0 ib/p/n) and Soe (0.8 ib/p/n), respectively (S. Dadzie, M. Appawu, personal communication). These substantial drops in HBR and EIR can be most likely be attributed to the three-rounds of IRS with organophosphates as no other vector control interventions were implemented in Bongo besides long-lasting insecticidal nets (LLINs), which were already widely available prior to the start of this study.

Var g**enotyping and estimated multiplicity of infection (MOI)**. The hyper-diverse multi-copy *var* gene family encodes the major variant surface antigen of the *P. falciparum* blood stage, called *P. falciparum* erythrocyte membrane protein (*Pf*EMP1). *Pf*EMP1 is the target of naturally acquired immunity to *P. falciparum* and is encoded by ~50-60 *var* genes per genome (Biggs et al., 1991; Gardner et al., 2002; Kyes, Kraemer, & Smith, 2007; Miller, Baruch, Marsh, & Doumbo, 2002; Scherf, Lopez-Rubio, & Riviere, 2008; Smith et al., 1995). The conserved DBLα domain of *var* genes was amplified from gDNA of the pre-IRS (T1) and post-IRS (T2) isolates as described by He et al., (2019) (He et al., 2018) with modifications as described by Ruybal-

Pesántez et al., (2017) (Ruybal-Pesántez et al., 2017). *Var* genotyping was used to infer MOI based on a conservative threshold of 60 unique *var* DBLα types per genome (i.e., isolates with ≤ 60 DBLα types were considered single-clone infections). The DBLα sequences and MOI data for the pre- and post-IRS isolates is published in Pilosof et al., (2019) (Pilosof et al., 2019). This *var* genotyping procedure provided a higher resolution to discern and select those asymptomatic *P. falciparum* isolates with an MOI ≤ 2.

Genetic differentiation. G_{ST} was historically thought to be indicative of genetic differentiation, but recent studies have supported that Jost's *D* values are a more accurate and robust measure (Gerlach, Jueterbock, Kraemer, Deppermann, & Harmand, 2010; Hedrick, 2005; Jost, 2008) (Jost 2008; Hedrick 2005; Gerlach et al., 2010). In fact, Jost et al. (2018) (Jost et al., 2018) posited that G_{ST} is a measure of the extent an allele is fixed between population; while Jost's *D* can be used for more than one allele per locus and calculates the allelic differentiation between populations. Jost's *D* varies from locus to locus as its value depends on the mutation rate of that locus. Jost's *D* weighs alleles according to the square of their relative frequencies and therefore measures the differentiation of the most common alleles. Jost's *D* is independent of within-group diversity and is more robust to variations in population size. A high value of Jost's *D* indicates that the most common alleles are not shared.

REFERENCES.

Biggs, B. A., Gooze, L., Wycherley, K., Wollish, W., Southwell, B., Leech, J. H., & Brown, G. V. (1991). Antigenic variation in Plasmodium falciparum. *PNAS*, *88*, 9171–9174.

Evanno, G., Regnaut, S., & Goudet, J. (2005). Detecting the number of clusters of individuals using the software STRUCTURE: a simulation study. *Molecular Ecology*, *14*, 2611–2620.

Gardner, M. J., Hall, N., Fung, E., White, O., Berriman, M., Hyman, R. W., … Barrell, B. (2002). Genome sequence of the human malaria parasite Plasmodium falciparum. *Nature*, *419*(6906), 498–511. https://doi.org/10.1038/nature01097

Gerlach, G., Jueterbock, A., Kraemer, P., Deppermann, J., & Harmand, P. (2010). Calculations of population differentiation based on GST and D: Forget GST but not all of statistics! *Molecular Ecology*, *19*(18), 3845–3852. https://doi.org/10.1111/j.1365-294X.2010.04784.x

He, Q., Pilosof, S., Tiedje, K. E., Ruybal-Pesántez, S., Artzy-Randrup, Y., Baskerville, E. B., … Pascual, M. (2018). Networks of genetic similarity reveal non-neutral processes shape strain structure in Plasmodium falciparum. *Nature Communications*, *9*(1), 1817. https://doi.org/10.1038/s41467-018-04219-3

Hedrick, P. W. (2005). A standardized genetic differentiation measure. *Evolution; International Journal of Organic Evolution*, *59*(8), 1633–1638. https://doi.org/Doi 10.1554/05-076.1

Jost, L. (2008). G(ST) and its relatives do not measure differentiation. *Molecular Ecology*, *17*(18), 4015–4026. https://doi.org/10.1111/j.1365-294X.2008.03887.x

Jost, L., Archer, F., Flanagan, S., Gaggiotti, O., Hoban, S., & Latch, E. (2018). Differentiation measures for conservation genetics. *Evolutionary Applications*, *11*(7), 1139–1148. https://doi.org/10.1111/eva.12590

Kyes, S. A., Kraemer, S. M., & Smith, J. D. (2007). Antigenic variation in Plasmodium falciparum: Gene organization and regulation of the var multigene family. *Eukaryotic Cell*, *6*, 1511–1520. https://doi.org/10.1128/EC.00173-07

Miller, L. H., Baruch, D. I., Marsh, K., & Doumbo, O. K. (2002). The pathogenic basis of malaria. *Nature*, *415*(6872), 673–679. https://doi.org/10.1038/415673a

Pilosof, S., He, Q., Tiedje, K. E., Ruybal-Pesántez, S., Day, K. P., & Pascual, M. (2019). Competition for hosts modulates vast antigenic diversity to generate persistent strain structure in Plasmodium falciparum. *PLOS Biology*, *17*(6), e3000336. https://doi.org/10.1371/journal.pbio.3000336

Ruybal-Pesántez, S., Tiedje, K. E., Tonkin-Hill, G., Rask, T. S., Kamya, M. R., Greenhouse, B., … Day, K. P. (2017). Population genomics of virulence genes of Plasmodium falciparum in clinical isolates from Uganda. *Scientific Reports*, *7*(1), 11810. https://doi.org/10.1038/s41598-017-11814-9

Scherf, A., Lopez-Rubio, J. J., & Riviere, L. (2008). Antigenic variation in Plasmodium falciparum. *Annual Review of Microbiology*, *62*(2), 445–470. https://doi.org/10.1146/annurev.micro.61.080706.093134

Smith, J. D., Chitnis, C. E., Craig, A. G., Roberts, D. J., Hudson-Taylor, D. E., Peterson, D. S., … Miller, L. H. (1995). Switches in expression of plasmodium falciparum var genes correlate with changes in antigenic and cytoadherent phenotypes of infected erythrocytes. *Cell*, *82*(1), 101–110. https://doi.org/10.1016/0092-8674(95)90056-X
